# The pivotal role of micro-environmental cells in a human blood–brain barrier in vitro model of cerebral ischemia: functional and transcriptomic analysis

**DOI:** 10.1186/s12987-020-00179-3

**Published:** 2020-03-05

**Authors:** Anna Gerhartl, Nadja Pracser, Alexandra Vladetic, Sabrina Hendrikx, Heinz-Peter Friedl, Winfried Neuhaus

**Affiliations:** Competence Unit Molecular Diagnostics, Center Health and Bioresources, AIT–Austrian Institute of Technology GmbH, Giefinggasse 4, 1210 Vienna, Austria

**Keywords:** Brain endothelial cells, Claudin, Cerebral ischemia, Stroke, Traumatic brain injury

## Abstract

**Background:**

The blood–brain barrier (BBB) is altered in several diseases of the central nervous system. For example, the breakdown of the BBB during cerebral ischemia in stroke or traumatic brain injury is a hallmark of the diseases’ progression. This functional damage is one key event which is attempted to be mimicked in in vitro models. Recent studies showed the pivotal role of micro-environmental cells such as astrocytes for this barrier damage in mouse stroke in vitro models. The aim of this study was to evaluate the role of micro-environmental cells for the functional, paracellular breakdown in a human BBB cerebral ischemia in vitro model accompanied by a transcriptional analysis.

**Methods:**

Transwell models with human brain endothelial cell line hCMEC/D3 in mono-culture or co-culture with human primary astrocytes and pericytes or rat glioma cell line C6 were subjected to oxygen/glucose deprivation (OGD). Changes of transendothelial electrical resistance (TEER) and FITC-dextran 4000 permeability were recorded as measures for paracellular tightness. In addition, qPCR and high-throughput qPCR Barrier chips were applied to investigate the changes of the mRNA expression of 38 relevant, expressed barrier targets (tight junctions, ABC-transporters) by different treatments.

**Results:**

In contrast to the mono-culture, the co-cultivation with human primary astrocytes/pericytes or glioma C6 cells resulted in a significantly increased paracellular permeability after 5 h OGD. This indicated the pivotal role of micro-environmental cells for BBB breakdown in the human model. Hierarchical cluster analysis of qPCR data revealed differently, but also commonly regulated clustered targets dependent on medium exchange, serum reduction, hydrocortisone addition and co-cultivations.

**Conclusions:**

The co-cultivation with micro-environmental cells is necessary to achieve a functional breakdown of the BBB in the cerebral ischemia model within an in vivo relevant time window. Comprehensive studies by qPCR revealed that distinct expression clusters of barrier markers exist and that these are regulated by different treatments (even by growth medium change) indicating that controls for single cell culture manipulation steps are crucial to understand the observed effects properly.

## Background

Cerebral ischemic insults are an immense burden for the national health care systems. Stroke is estimated to cost the EU economy €45 billion a year, whereby 44% (€20 billion) is due to direct health care costs, 22% (€9 billion) to productivity losses and 35% (€16 billion) to the informal care of people with stroke [51]. The total European annual health care cost of traumatic brain injury (TBI) is over €33 billion [2].

The blood–brain barrier (BBB) plays an important role in both clinical pictures (stroke, TBI). The reduced supply of oxygen and nutrients such as glucose causes damage of the BBB, which contributes to the development of brain edema. After reperfusion, damage to the BBB occurs in several phases, whereby it is assumed that after several hours, an increase in the transcytosis rate is initially observed, followed by a break-up of the paracellular tight junctions after 1 and 2 days [20]. Investigations in even shorter periods of time showed that an opening and dysfunction of the BBB can already occur within 30–45 min after the insult [41]. The BBB has the task to protect the central nervous system (CNS) from physical, chemical and biological damage and to maintain homeostasis within the CNS. The main sealing component of the BBB are the brain capillary endothelial cells (BCECs). They are characterized by very tight cell–cell junctions and a battery of transporter proteins and enzymes that enable them to build a physical, a transport and a metabolic barrier. In contrast to peripheral endothelial cells, BCECs have hardly fenestrae, a significantly reduced pinocytosis rate and a significantly higher mitochondrial density to provide the energy for maintaining barrier function [18]. The paracellular gap is sealed by tight junctions and this prevents the uncontrolled transport of hydrophilic molecules. Currently, the presumably most important tight junction (TJ) proteins of the BBB are the sealing claudins-1, -3, -5, -11 and -12 and the TJ structure regulating proteins occludin, tricellulin, LSR and zonula occludens 1 (ZO-1). At the moment, however, there is a lively discussion about the role of individual claudins at the BBB. For example, it was recently shown that the claudins-3 and -12 in the BBB of the mouse do not have the previously believed importance for BBB function [7, 8]. In addition, there are postulated species differences that make it difficult to get a clear picture of which claudins are present in the human BBB and which function they fulfill [3]. Previous studies have in common that they prove the essential role of claudin-5 in all species. However, in vivo tissue and in vitro data from recent publications indicate that the TJ claudin network at the BBB may be much more complex than previously assumed [11, 24, 47]. To maintain the transport barrier, the BCECs use an array of transporter proteins, most of which belong to either the ABC or the SLC transporter families. According to current knowledge, ABCB1 (P-gylcoprotein), ABCG2 (BCRP, breast cancer resistance protein) and ABCC1-5 (MRP1-5, multidrug-resistance related proteins) are mainly responsible for preventing the entry into the CNS of undesirable substances such as xenobiotics and/or drugs [33]. The function of both the TJs and the ABC transporters on the BBB is strictly regulated by the microenvironment. The closest cells to the BCECs on the CNS side are the pericytes, which even share the basement membrane with the BCECs, and the astrocytes, whose terminal endfeet cover up to 90% of the capillary surface on the CNS side. Astrocytes are thought to induce BBB properties such as the paracellular barrier or ABC transporter activities, whereas pericytes suppress peripheral endothelial cell properties in BCECs such as the significantly higher peripheral pinocytosis rate [10]. In the case of cerebral ischemia, the BBB is damaged within a few hours, whereby the TJ lose their integrity and some ABC transporters are regulated to protect the cells [5, 17, 28, 41]. This loss of function can be measured by increased entry of permeability markers into the CNS. In vitro, this can also be determined non-invasively by measuring the reduction of the transendothelial electrical resistance (TEER). During cerebral ischemia, glucose transporters such as SLC2A1 (Glut-1) are upregulated, by which BCECs try to take up the remaining glucose for stabilizing the energy balance. Like the vascular endothelial growth factor (VEGF), this upregulation is hypoxia-dependent and can also be used as a marker for the reaction of BCECs to hypoxic states [30, 52]. Although a large number of research projects were carried out on novel therapies for the treatment of cerebral ischemia, clinical success to date has been marginal. Several reports showed that the stabilization of the BBB during acute insults can lead to a significant reduction in brain edema and neurological damage [19, 32, 43]. Nevertheless, the mechanisms during cerebral ischemia are not yet sufficiently understood. In addition, the existing studies were mostly performed in rodents both in vivo and in vitro. However, to ensure a useful translation to the human situation, well validated human in vitro models of the BBB for cerebral ischemia are required. It is noticeable that the incubation times applying OGD (oxygen/glucose deprivation as treatment to simulate cerebral ischemia) to detect functional damage in in vitro models were often much longer (up to 24 h) than in in vivo models or known from the clinic [36, 37]. In this context, it was shown in in vitro mouse models that co-cultivation with micro-environmental cells such as astrocytes or glioma cells significantly shortened incubation times to more in vivo relevant durations while achieving the same functional damage [29, 31]. Therefore, one major objective of this study was to establish a human in vitro BBB model of cerebral ischemia that achieves a functional damage of approximately 35–60% TEER decline in less than 6 h. This TEER decline was defined in previous mouse in vitro studies as optimal for therapy testing, as too little damage makes the read-out of positive effects difficult and too much damage shows insufficient reversibility [31, 32]. The other major objective was to investigate comprehensively the influences of experimental parameters on the expression of TJ proteins and ABC transporters. As human in vitro BBB model, the most commonly used human cell line hCMEC/D3 [49] was cultivated and co-cultured with rat glioma cell line C6 or primary human astrocytes/pericytes under normoxic and OGD conditions. Cell line C6 was chosen, since their usability for inducing BBB breakdown has already been proven in a mouse ischemia model [31, 32].

## Methods

### Cell culture

The human immortalized cell line hCMEC/D3 [50] was obtained from Merck Milipore, Darmstadt, Germany (Ref.: SCC066) and cultured on 0.5% gelatin-coated culture flasks (Gelatin: SERVA Electrophoresis GmbH, Heidelberg, Germany; 22,151.02; culture flasks: CellStar, Greiner Bio-one, Kremsmünster, Austria; 690175 or 658175) in EBM-2 (Lonza, Basel, Swiss; CC 3156) supplemented with 5% Fetal Calf Serum (FCS; Sigma-Aldrich, St. Louis, USA; F9665), 1% penicillin/streptomycin (Biochrom GmbH, Berlin, Germany; A2213) as well as 10 mM HEPES (Sigma-Aldrich, St. Louis, USA; H0887), 5 µg/mL ascorbic acid (Sigma-Aldrich, St. Louis, USA; A4544-25G) and 1 ng/mL hbFGF (Sigma-Aldrich, St. Louis, USA; F0291-25UG). For maintenance hCMEC/D3 were treated with 0.25% trypsin/EDTA (Biochrom GmbH, Berlin, Germany; L2143) for 3–5 min at 37 °C and subcultivated in a ratio of 1:3 once a week. Human primary astrocytes (hA; Provita AG, Germany; SC-1800-5) and human primary pericytes (hP; Provita AG, Germany; SC-1200) were cultured on 10 µg/mL Poly-l-Lysine (ScienCell, Carlsbad, USA; 413) coated culture flasks in either astrocyte medium AM (ScienCell, Carlsbad, USA; 1801) supplemented with 2% FCS (ScienCell, Carlsbad, USA; sc-0010), 1% of penicillin/streptomycin (ScienCell, Carlsbad, USA, sc-0503) and 1% astrocyte growth supplement (ScienCell, Carlsbad, USA; sc-1852) or pericyte medium PM (ScienCell, Carlsbad, USA; sc-1201) supplemented with 2% FCS (ScienCell, Carlsbad, USA; 0010), 1% of penicillin/streptomycin (ScienCell, Carlsbad, USA; sc-0503) and 1% pericyte growth supplement (ScienCell, Carlsbad, USA; sc-1252) respectively. For subcultivation hA and hP were treated with Accutase (Sigma-Aldrich, St. Louis, USA; A6964-100ML) for 2–3 min at 37 °C and seeded in a cell density of 6700 cells/cm^2^ [1]. The rat glioma cell line C6 was obtained from ATCC and kept in culture on 0.5% gelatin-coated culture flasks in high-glucose DMEM (Sigma-Aldrich, St. Louis, USA; D5796) supplemented with 10% FCS (Sigma-Aldrich, St. Louis, USA; F9665) and 1% penicillin/streptomycin (Biochrom GmbH, Berlin, Germany; A2213). For subcultivation C6 were treated with 0.25% trypsin/EDTA (Biochrom GmbH, Berlin, Germany; L2143) for 1–3 min at 37 °C until detachment and re-seeded in a ratio of 1:20. All cell types were kept in an incubator (Thermo Fisher Scientific HERACell Vios 160i CO_2_ Incubator) at 37 °C in 5% CO_2_/95% air atmosphere and 95% humidity.

### Oxygen/glucose deprivation treatment

**Fig. 1:**
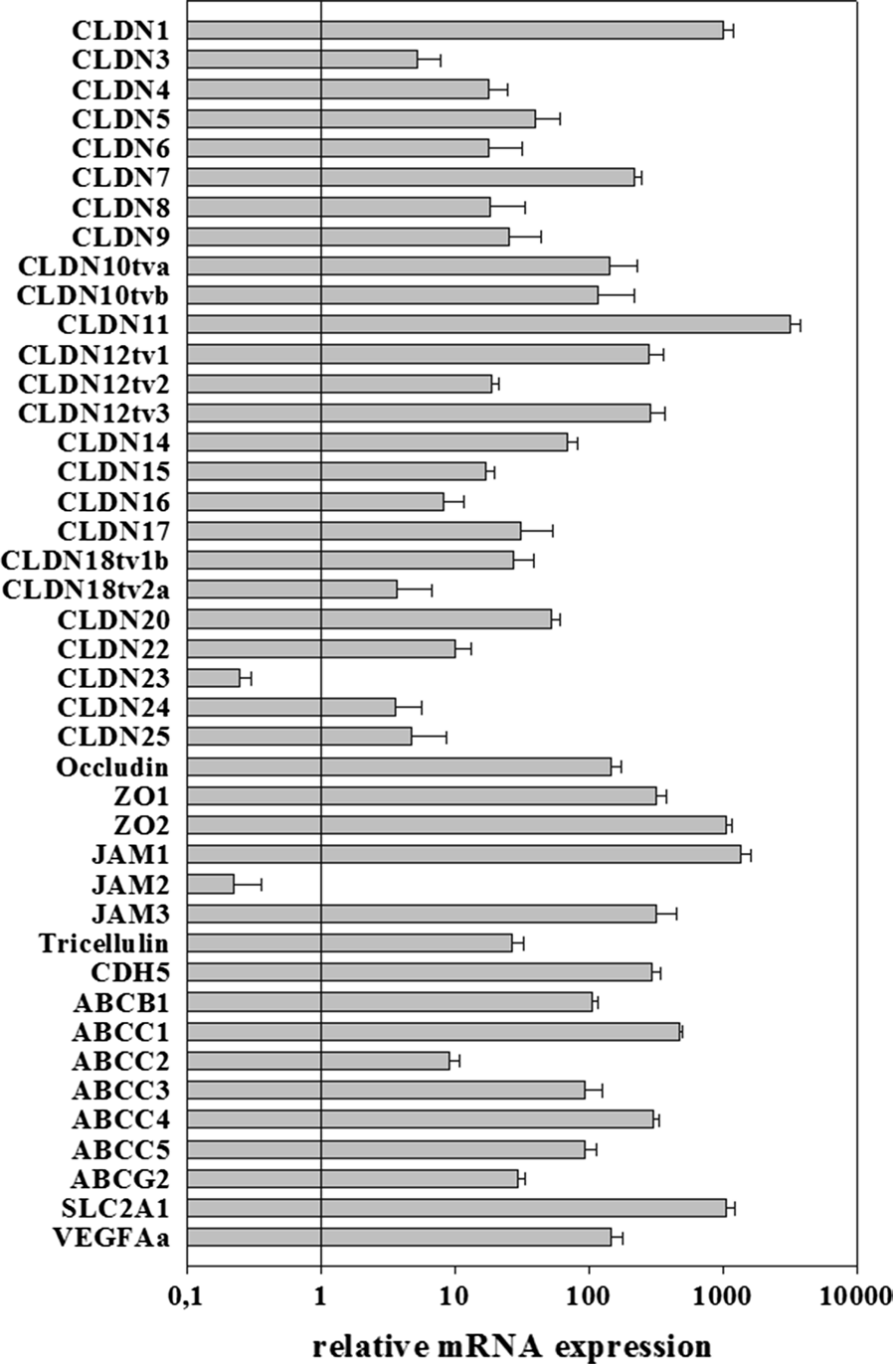
Expression levels of tight junction proteins, ABC-transporters, VE-cadherin (CDH5), SLC2A1 (GLUT-1) and VEGFa in hCMEC/D3 cells cultivated in EBM-2 medium with 5% FCS for 5 days and incubated in EBM-2 medium with a reduced serum amount of 0.25% for additional 24 h from day 5 to day 6. After normalization to endogenous control b-actin and correction by individual primer efficacies, claudin-1 expression was set to 1000. Data are presented as mean ± SEM, n = 4 from four independent experiments

**Fig. 2:**
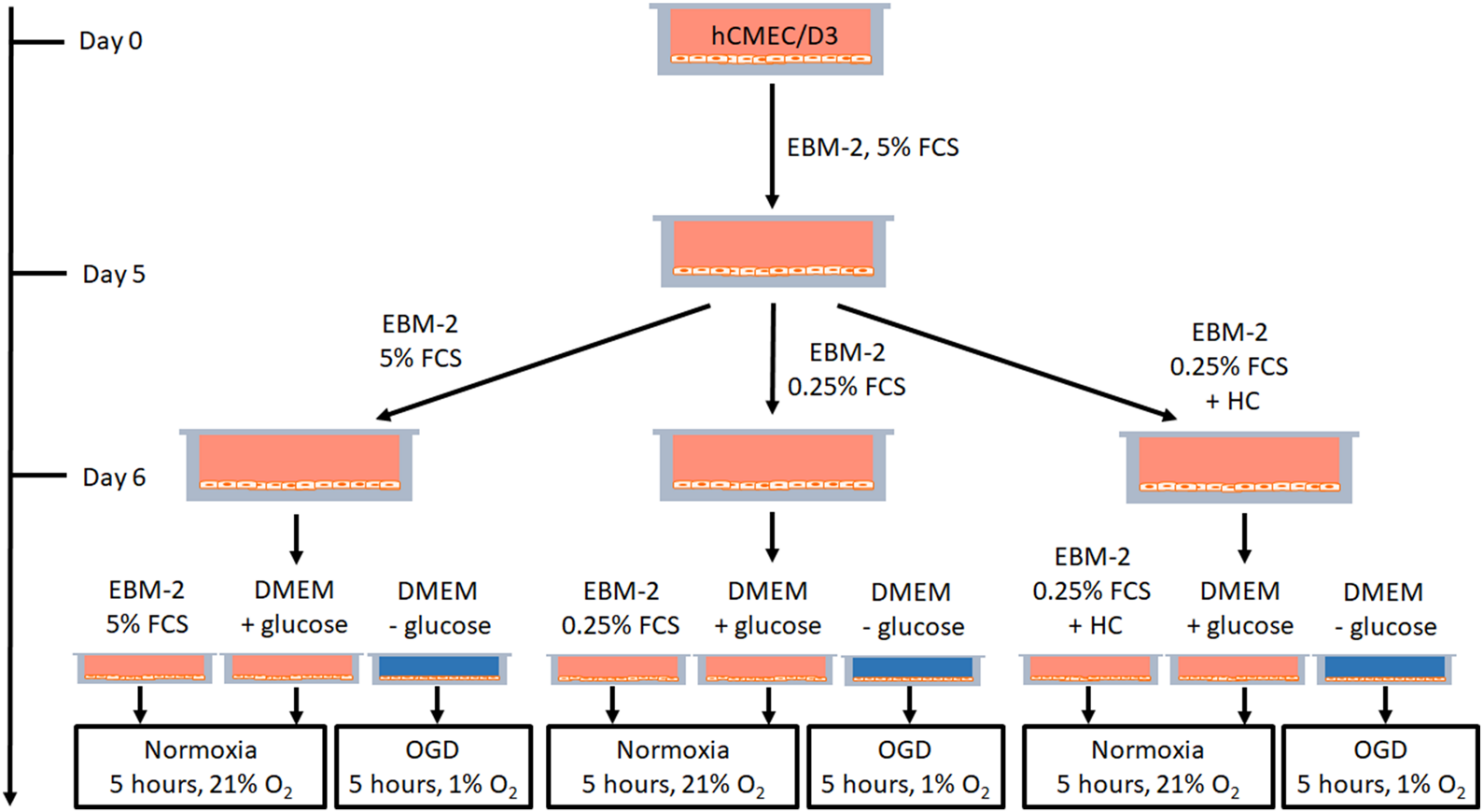
Experimental treatment scheme for the hCMEC/D3 medium dependent OGD experiments conducted in 6-well plates, *FCS* fetal calf serum, *OGD* oxygen/glucose deprivation, *HC* hydrocortisone

**Fig. 3:**
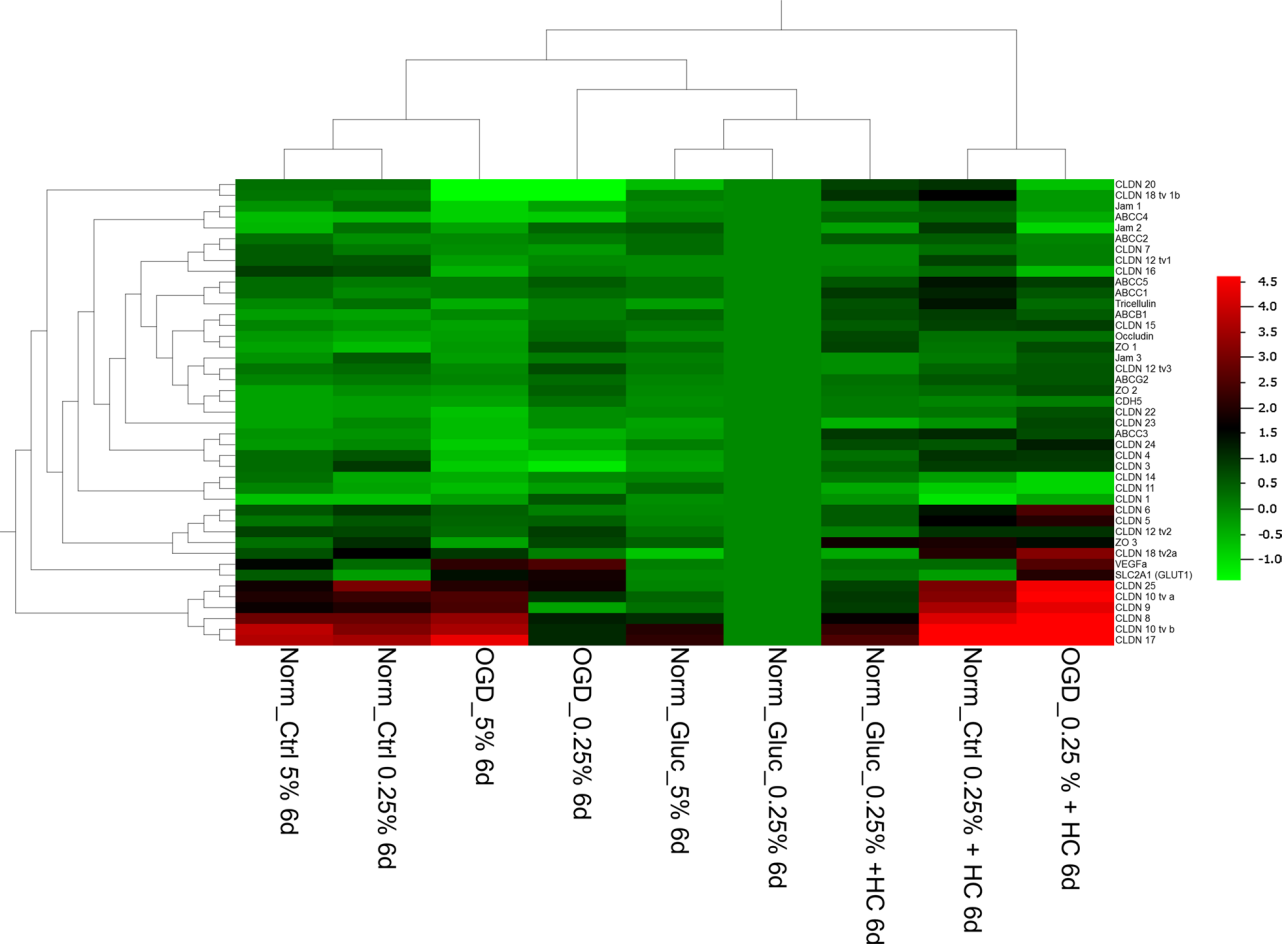
Hierarchical clustering of mRNA expression x-fold data normalized to the mean value (from n = 8 from four independent experiments) with of each single target from hCMEC/D3 cells cultivated for 5 days in EBM-2 medium with 5% FCS, subjected to 24 h serum reduction from 5 to 0.25% FCS and were cultivated on day 6 for 5 h under normoxic conditions in DMEM medium with glucose. Norm_Ctrl = 5 h in normoxia in EBM-2 medium on day 6, Norm_Gluc = 5 h in normoxia in DMEM medium containing glucose, OGD = 5 h in DMEM glucose-free medium in the hypoxia chamber at 1% O_2_, 5% 6d = cells remained in medium supplemented with 5% FCS from day 5 to day 6, 0.25% 6d = cells underwent serum reduction from 5 to 0.25% FCS in the according medium, HC = addition of 100 nM hydrocortisone from day 5 to day 6. The colour code legend is formatted in log-scale

For Figs. 1 and 3 hCMEC/D3 cells were seeded onto 0.5% gelatin-coated 6-well plates (Falcon, BD Biosciences, Franklin Lakes, USA; 353 502) at a density of 40,000 cells/cm^2^. Medium change was performed every other day. On day 5, the serum concentration of EBM-2 was optionally reduced from 5% to 0.25% FCS or 0.25% FCS with 100 nM hydrocortisone for 24 h. On day 6, oxygen/glucose deprivation (OGD) was performed. Hypoxic conditions were established by changing the medium to DMEM without glucose and without serum (Gibco^®^, Thermo Fisher Scientific, Waltham, USA; 11966-025) and by reducing the O_2_ level to 1% by placing the plate into a biospheryx chamber (37 °C, 5% CO_2_; Biospheryx, USA) for 5 h. As normoxia controls, the cells were kept in EBM-2 with 5 or 0.25% FCS or were cultivated in DMEM with glucose (Gibco^®^, Thermo Fisher Scientific, Waltham, USA; 31966-021) but without serum for 5 h in an incubator (37 °C, 5% CO_2_). The experimental treatment scheme is presented in Fig. 2. After 5 h, cell lysis was conducted with RA-1 buffer containing 1% β-Mercaptoethanol (Sigma-Aldrich, St. Louis, USA, 913148-250ML) according to manual instructions of the NucleoSpin^®^ RNA kit (Macherey–Nagel, Düren, Germany; 740955.250). RNA samples were stored at − 80 °C until RNA isolation.

**Fig. 4:**
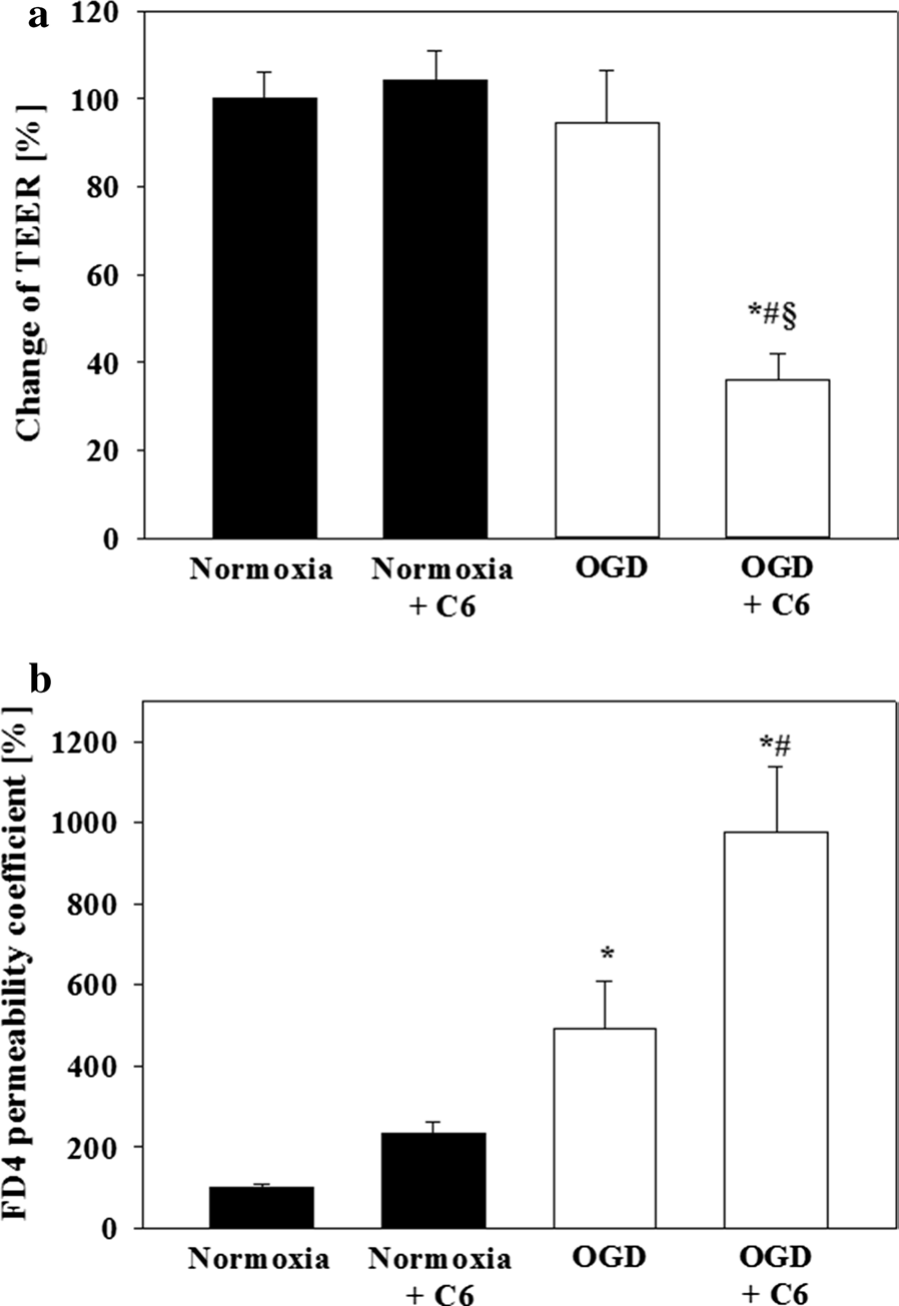
Addition of glioma C6 cells increased the damage of the paracellular barrier of hCMEC/D3 layers after 5 h OGD treatment at 1% O_2_. **a** TEER change is normalized to the TEER progression of the normoxia control. **b** Permeability of FITC-dextran 4000 (n = 9–12 inserts from three independent experiments). Data are presented as mean ± SEM. *Statistical significant versus normoxia hCMEC/D3, *p *< 0.05, ^#^statistical significant versus OGD hCMEC/D3 + C6, *p *< 0.05; ^§^statistical significant versus normoxia hCMEC/D3, *p *< 0.05. Two-Way ANOVA with all pairwise multiple comparison Holm-Sidak in case of non-normality distribution or non-equal variances

**Fig. 5:**
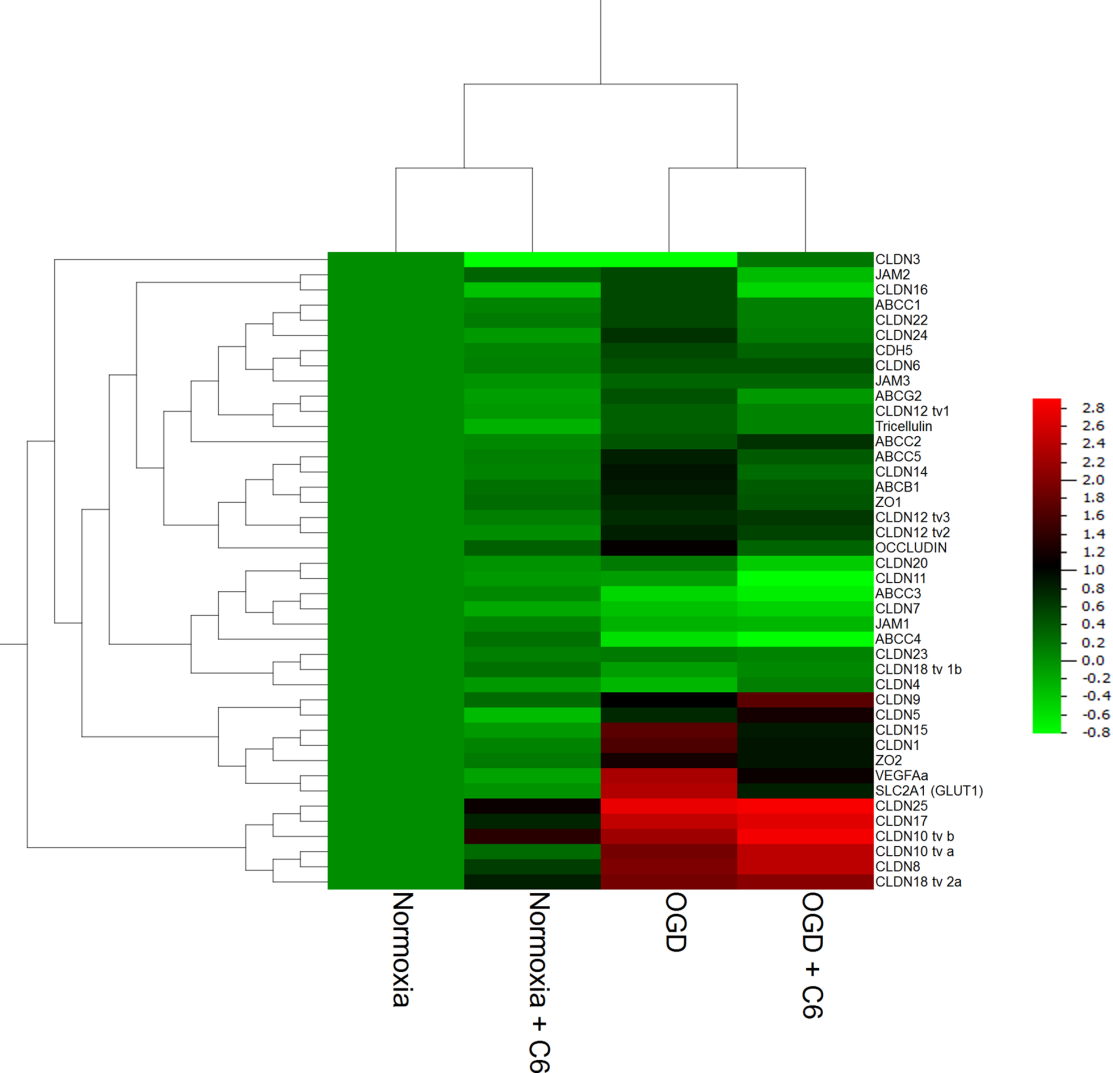
Hierarchical clustering of mRNA expression x-fold data normalized to the mean value (n = 3 from three independent experiments, pooling of four replicates in each single experiment was performed) of each single target from hCMEC/D3 cells incubated under normoxia or OGD conditions (1% O_2_) as mono- or co-culture with glioma C6 cells for 5 h. The colour code legend is formatted in log-scale

**Fig. 6:**
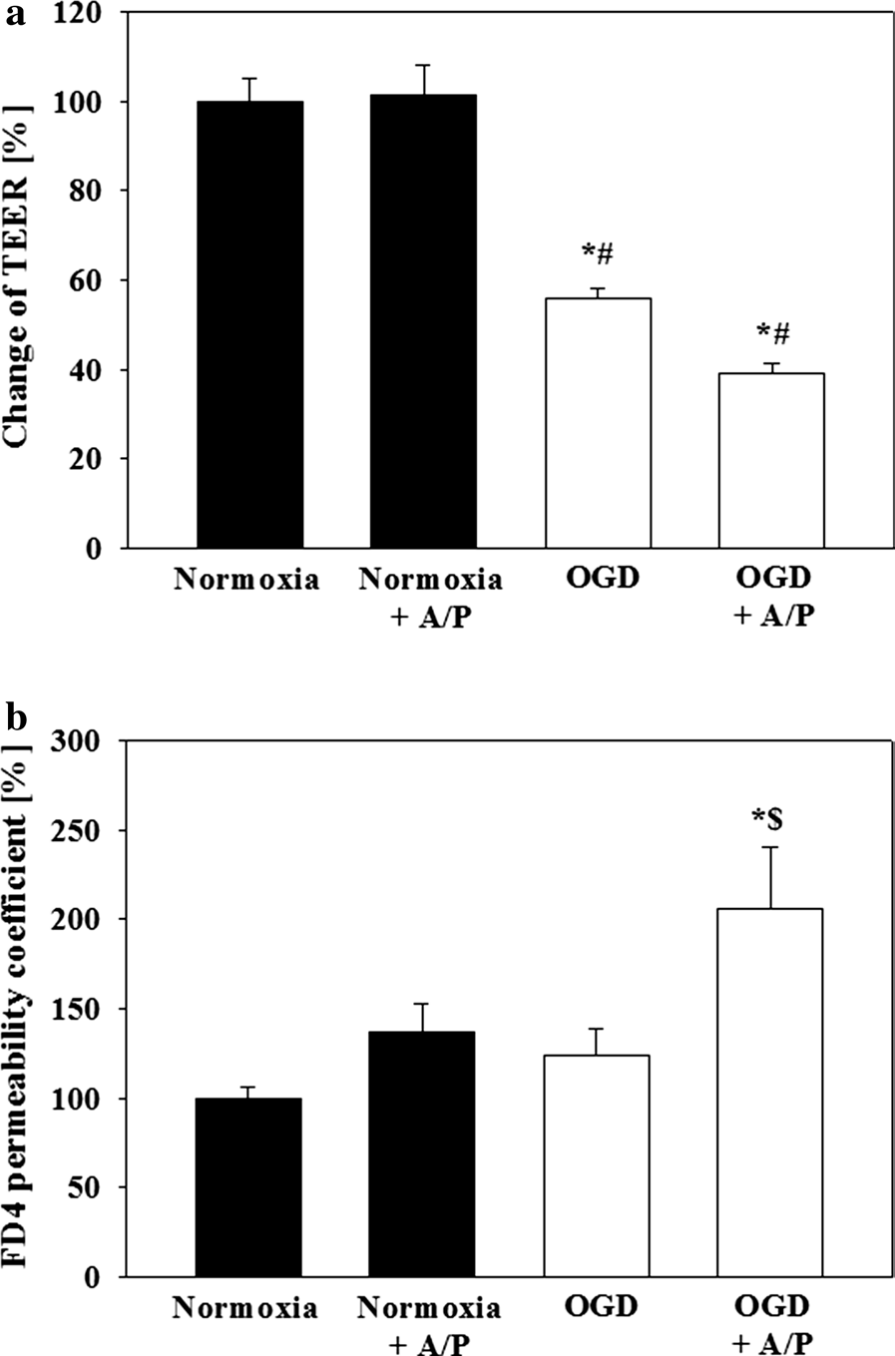
Addition of a 1:1 mixture of human primary astrocytes and pericytes increased the damage of the paracellular barrier of hCMEC/D3 layers after 5 h OGD treatment at 0.1% O_2_. **a** TEER change is normalized to the TEER progression of the normoxia control. **b** Permeability of FITC-dextran 4000 (n = 12 inserts from three independent experiments). Data are presented as mean ± SEM. *Statistical significant versus normoxia hCMEC/D3, *p *< 0.05, ^#^Statistical significant versus OGD hCMEC/D3, *p *< 0.05; ^§^statistical significant versus normoxia hCMEC/D3 + hA/hP, *p *< 0.05. Two-Way ANOVA with all pairwise multiple comparison Holm-Sidak in case of non-normality distribution or non-equal variances

**Fig. 7:**
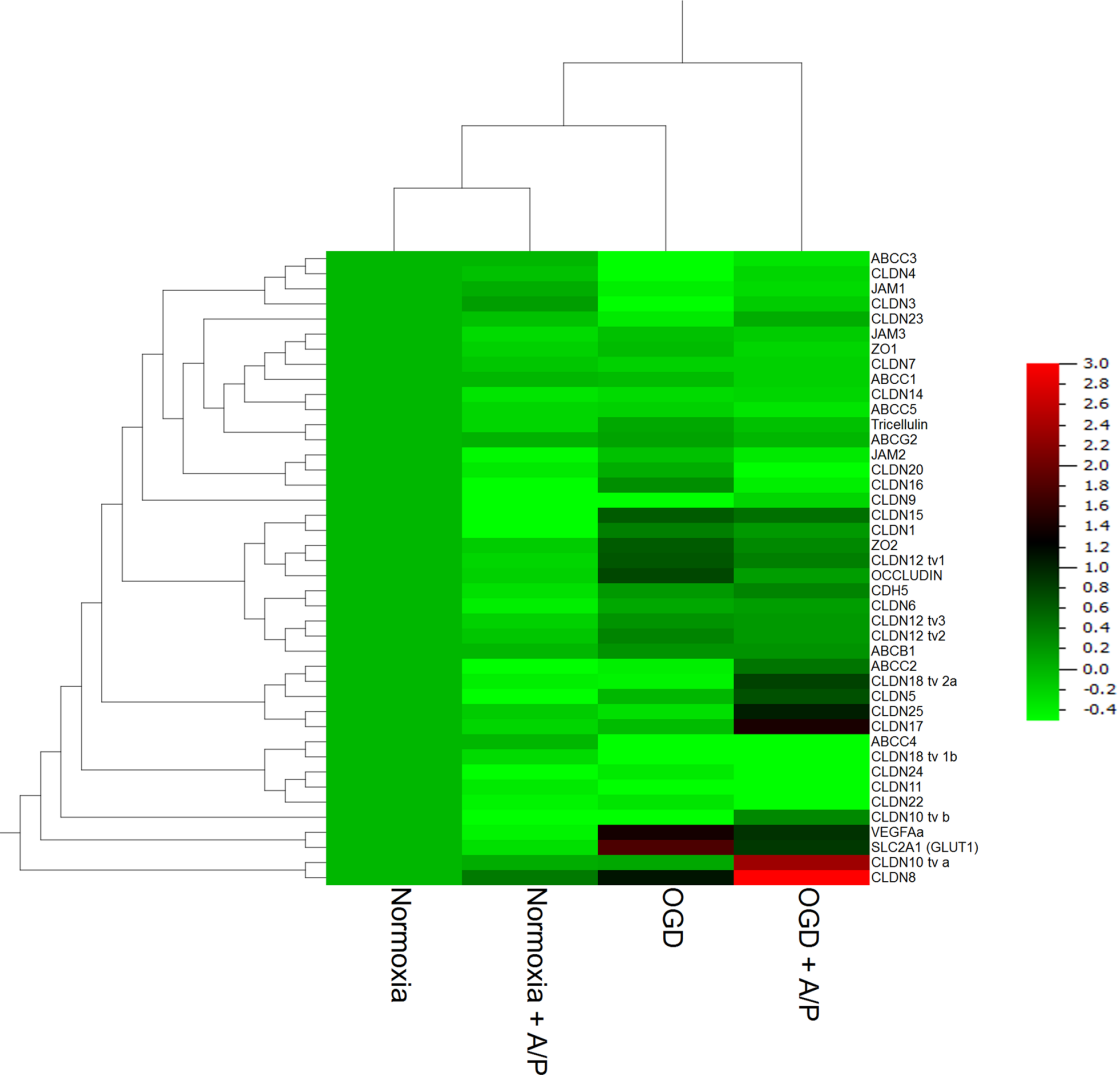
Hierarchical clustering of mRNA expression x-fold data normalized to the mean value of each single target from hCMEC/D3 cells incubated under normoxia or OGD conditions (0.1% O_2_) as mono- or co-culture with a 1:1 mixture of human primary astrocytes and pericytes for 5 h. The colour code legend is formatted in log-scale

For Figs. 4, 5, 6, 7 Transwell experiments were conducted based on recent publications [31, 34]. Briefly, hCMEC/D3 were seeded in a density of 80,000 cells/cm^2^ onto four 24-well inserts per condition and experiment (Costar Corning, Corning, USA; 3470, pore size: 0.4 µm, polyester membrane) coated with a mixture of collagen IV (0.1 mg/mL; Sigma-Aldrich, St. Louis, USA; C5533) and fibronectin (1 mg/mL; Sigma-Aldrich, St. Louis, USA; F1141-5MG) in sterile Millipore water or PBS (Thermo Fisher Scientific, Waltham, USA; 14190094). For co-cultures, human primary astrocytes (hA) and human primary pericytes (hP) respectively the rat glioma cell line C6 were cultivated in 24-well plates either coated with Poly-l-Lysine (P-l-L; ScienCell, Carlsbad, USA; 0413) for hA and hP or 0.5% gelatin for C6 with a cell density of 25.000 cells/cm^2^ each for hA and hP and a cell number of 20,000 cells/cm^2^ for C6. hA and hP were seeded 3 days, C6 were seeded 6 days prior experiment conduction. Medium was changed every other day. On day 6 after seeding the hCMEC/D3, the hCMEC/D3 were put into co-culture with hA and hP or C6 and were exposed to OGD for barrier breakdown induction. Hence, the mono- and co-cultures were treated with DMEM without glucose and without serum and were placed in the biosheryx chamber (37 °C, 5% CO_2_) with controlled O_2_ level of 0.1% (hAP) respectively 1% (C6) for 5 h. As control in normoxic condition, mono- and co-cultures were cultivated in DMEM with glucose but without serum for 5 h in an incubator with atmospheric O_2_ levels (37 °C, 5% CO_2_). After 5 h treatment, barrier breakdown was assessed by TEER (transendothelial electrical resistance) measurements with a chopstick electrode (Milipore, Burlington, USA) after a 30 min RT equilibration period and by permeability studies with the paracellular marker fluorescein isothiocyanate-dextran (10 µM; FD4; 4 kDa; Sigma-Aldrich, St. Louis, USA; FD4-250MG, 1 mM stock solution of FD4 ultra-filtered with Amicon tubes with a cut-off 3 kDa to separate from residual, free FITC). The actual TEER values [Ω cm^2^] were determined by substracting the mean value of the blank values multiplied by the growth surface area of 0.336 cm^2^. Data were normalized to normoxia controls and expressed in [%]. The FD4 permeability coefficient was calculated as previously published according the clearance principle substracting the permeability of the blank inserts without cells to obtain the permeability coefficient of the cell layers only [34]. Additionally, cells were lysed after the experiments in RA-1 buffer and samples of the same treatment were pooled (i.e. all four inserts per treatment) for subsequent mRNA analysis.

### Real-time quantitative PCR (qPCR)

Samples for qPCR analysis were generated by harvesting hCMEC/D3 cultivated on either 6-well plates or on 24-well inserts in mono- or co-culture after experiment conduction and physical barrier integrity assessment. RNA isolation was performed with the NucleoSpin RNA Kit according to manufacturer’s instructions (Machery-Nagel, Düren, Germany; Ref.: 740.955.250). For cDNA synthesis either 250 ng or 1 µg RNA was reversely transcribed into 20 µL total volume using the High Capacity cDNA Reverse Transcription Kit (Applied Biosystems/Thermo Fisher Scientific; Ref.: 4368814). For Figs. 4 and 6 qPCR was conducted with the PowerUp SybrGreen MasterMix (Applied Biosystems/Thermo Fisher Scientific; Ref.: A2 5742) and a primer dilution of 1:33. The LightCycler^®^480 (Roche Applied Science, Basel, Switzerland) was programmed as following: Holding Stage 95 °C for 20 s, 40 cycles at 95 °C for 3 s and 60 °C for 30 s; Melting Stage 95 °C for 15 s and 60 °C for 1 min followed by 95 °C for 15 s. For Figs. 1 and 3 the analysis was accomplished by high-throughput qPCR using 96 samples × 96 targets chips (Fluidigm^®^) as published recently [38]. The targets investigated included: PPIA, β-actin, GAPDH, B2M, claudin-1 to claudin-25, JAM-1 to JAM-3, ZO-1 to ZO-3, SCL2A1 (GLUT-1), VEGFa, VE-cadherin (CDH5), occludin, ABCB1, ABCG2, ABCC1-5 and tricellulin. All primers were validated by controlling the amplicon product sizes on agarose gels, assessing the melting curves after each qPCR run and determination of the primer efficiencies with at least four different cDNA concentrations. 2^ΔCt^-values were calculated and normalized to the endogenous housekeeping gene PPIA. Differences in mRNA level of the targets of interest are visualized in heat maps generated with the software Qlucore Omics Explorer 3.3.

### Statistics

Statistical analysis was performed by Two-Way ANOVA with all pairwise multiple comparison Holm-Sidak in case of non-normality distribution or non-equal variances using SigmaPlot 14. Hierarchical cluster analysis was performed with the Qlucore Omics Explorer 3.3.

## Results

### Human brain endothelial hCMEC/D3 cells express several tight junction and multidrug-resistant ABC transporter proteins

First the relative expression of claudins and ABC transporters in hCMEC/D3 cells was investigated under standard cultivation in EBM-2 medium with 5% FCS for 5 days followed by a serum reduction step in EBM-2 medium with 0.25% FCS for additional 24 h from day 5 to day 6. Interestingly, hCMEC/D3 expressed almost all claudins, only claudin-2 and -19 were not detected at the mRNA level (claudin-13 and -21 do not exist in human). Data were normalized to claudin-1 expression which was set to the value 1000 (Fig. 1, images of the agarose gels of the PCR products were summarized in Additional file 1). Compared to claudin-1 expression (1000), only claudin-11 (3189), ZO-2 (1065), JAM-1 (1347) and SLC2A1 (1065) showed a higher abundance. The ranking order for the claudins is listed in Table 2. In case of the ABC transporters the highest abundant was ABCC1 (478) followed by ABCC4 (299), ABCB1 (106), ABCC3 (93), ABCC5 (92), ABCG2 (29) and ABCC2 (9). Quite high and moderate expression was found for JAM3 (320), ZO-1 (315), CDH5 (294) and Occludin (145), whereas low expression was found for Tricellulin (27) and JAM-2 (0.23).

### Medium exchange, serum reduction, hydrocortisone and oxygen/glucose deprivation alter the target expressions in hCMEC/D3 cells

Since serum reduction and addition of hydrocortisone were published to increase paracellular tightness of hCMEC/D3 cells [14], it was decided to investigate the influence of these treatments—in our case serum reduction from 5 to 0.25% FCS and the addition of 100 nM hydrocortisone—on the expression of the selected barrier targets. After these treatments from day 5 to day 6, cells were either changed to the same previous treatment media or to DMEM (+ glucose) or subjected to OGD (at 1% O_2_) in DMEM without glucose for 5 h (see scheme on Fig. 2). The data were normalized to the normoxia samples in DMEM (+ glucose, see Table 1). Comparison of these data revealed that the serum reduction under normoxia conditions led to a significant upregulation of JAM-1 from 0.91 ± 0.07 to 1.27 ± 0.11 and downregulation of ABCC2 from 1.22 ± 0.12 to 0.94 ± 0.02, SLC2A1 from 1.41 ± 0.16 to 0.85 ± 0.07 and VEGFa from 2.93 ± 0.50 to 1.26 ± 0.14 compared to EBM-2 with 5% FCS, whereas the addition of hydrocortisone at 0.25% FCS significantly decreased claudin-11 expression from 0.81 ± 0.05 to 0.58 ± 0.06 and increased ABCC3 from 0.91 ± 0.06 to 2.19 ± 0.16 and ABCC4 from 0.68 ± 0.02 to 1.33 ± 0.3 in comparison to 0.25% FCS normoxia. Several changes were induced by only changing the medium from EBM-2 with 0.25% FCS to DMEM (+ glucose) under normoxia conditions for 5 h. For example, claudin-1 (0.62 ± 0.07), claudin-11 (0.81 ± 0.05), ZO-1 (0.65 ± 0.06), occludin (0.78 ± 0.06), CDH5 (0.83 ± 0.03), ABCB1 (0.79 ± 0.06), ABCC4 (0.68 ± 0.02) and SLC2A1 (0.85 ± 0.07) were upregulated to 1.00 (normalized values of samples in DMEM plus glucose medium), whereas claudin-12 tv1 (1.48 ± 0.18), claudin-12 tv2 (1.72 ± 0.19) and JAM-1 (1.27 ± 0.11) were downregulated because of the growth medium change from EBM-2 (0.25% FCS) to DMEM (plus glucose). In addition, OGD increased expression of claudin-1 (1.52 ± 0.10), claudin-5 (1.35 ± 0.14), claudin-12 tv2 (1.89 ± 0.19), claudin-12 tv3 (1.63 ± 0.14), ZO-2 (1.39 ± 0.14), ZO-3 (1.71 ± 0.30), CDH5 (1.23 ± 0.09), ABCC1 (1.27 ± 0.09), ABCC5 (1.45 ± 0.15), SLC2A1 (3.62 ± 0.43) and VEGFa (5.63 ± 0.49) or decreased claudin-18 tva (0.31 ± 0.08), claudin-20 (0.38 ± 0.12), JAM-1 (0,80 ± 0.07), ABCC3 (0.72 ± 0.04) and ABCC4 (0.59 ± 0.06) when comparing cells cultured in EBM-2 with 0.25% FCS and changed into DMEM (+ glucose) for the normoxia and DMEM (without glucose) for the OGD treatment (further details in Table 1).

**Table 1:** Regulation of 38 barrier targets on the mRNA level in hCMEC/D3 samples, which were treated either with 5% FCS, 0.25% FCS or 0.25% FCS plus 100 nM hydrocortisone (HC) in EBM-2 medium prior OGD treatment

Day 5 on 6	5% EBM-2	0.25% EBM-2	0.25% EBM-2 + HC	5% EBM-2	0.25% EBM-2	0.25% EBM-2 + HC	5% EBM-2	0.25% EBM-2	0.25% EBM-2 + HC
5 h treatment	5% EBM-2	0.25% EBM-2	0.25% EBM-2 + HC	DMEM + Glu	DMEM + Glu	DMEM + Glu	DMEM–Glu	DMEM–Glu	DMEM–Glu
Normoxia/OGD	N	N	N	N	N	N	OGD	OGD	OGD
Claudin-1	0.63 ± 0.10*	0.62 ± 0.07*	0.46 ± 0.07*	0.94 ± 0.10	1.00 ± 0.00	0.92 ± 0.15	0.83 ± 0.07*^§^	1.52 ± 0.10*	0.77 ± 0.06*^§^
Claudin-3	1.25 ± 0.24	1.89 ± 0.88	1.83 ± 0.53	0.80 ± 0.14	1.00 ± 0.00	1.38 ± 0.32	0.58 ± 0.28	0.44 ± 0.11*	1.86 ± 0.93
Claudin-4	1.28 ± 0.24	1.49 ± 0.31	1.97 ± 0.42*	0.85 ± 0.08	1.00 ± 0.00	1.19 ± 0.17	0.66 ± 0.22	0.59 ± 0.07*	1.92 ± 0.89
Claudin-5	1.17 ± 0.26	1.51 ± 0.47	3.00 ± 0.72*	1.03 ± 0.11	1.00 ± 0.00	1.42 ± 0.30	1.31 ± 0.32	1.35 ± 0.14*	3.98 ± 1.40
Claudin-6	1.48 ± 0.49	1.93 ± 0.86	2.54 ± 0.98	0.97 ± 0.19	1.00 ± 0.00	1.43 ± 0.40	1.39 ± 0.70	1.07 ± 0.18	5.66 ± 3.09
Claudin-7	1.41 ± 0.22	1.14 ± 0.08	1.21 ± 0.08*	1.28 ± 0.15	1.00 ± 0.00	0.97 ± 0.11	0.96 ± 0.09	0.88 ± 0.10	1.08 ± 0.22
Claudin-8	7.28 ± 4.89	7.41 ± 4.06	18.02 ± 12.19	2.09 ± 1.76	1.00 ± 0.00	3.13 ± 1.81	10.05 ± 8.27	2.40 ± 0.95	53.64 ± 35.49
Claudin-9	3.25 ± 1.48	3.93 ± 2.04	12.19 ± 8.17	1.20 ± 0.60	1.00 ± 0.00	1.82 ± 0.63	5.44 ± 4.16	0.80 ± 0.16	20.00 ± 13.21
Claudin-10 tva	3.91 ± 2.46	4.77 ± 2.16	9.09 ± 5.88	1.31 ± 1.02	1.00 ± 0.00	1.96 ± 1.11	5.58 ± 4.60	1.96 ± 0.74	29.76 ± 19.82
Claudin-10 tvb	14.02 ± 8.92	8.74 ± 5.73	26.92 ± 18.18	4.12 ± 3.57	1.00 ± 0.00	4.53 ± 2.51	11.80 ± 10.29	2.21 ± 0.79	61.32 ± 40.30
Claudin-11	1.02 ± 0.13	0.81 ± 0.05*	0.58 ± 0.06^#^*	1.28 ± 0.18	1.00 ± 0.00	0.78 ± 0.05*	0.66 ± 0.05*	0.82 ± 0.09	0.54 ± 0.06*^§^
Claudin-12 tv1	1.43 ± 0.12*	1.48 ± 0.18*	1.78 ± 0.14*	0.98 ± 0.08	1.00 ± 0.00	1.00 ± 0.10	0.83 ± 0.05*	1.00 ± 0.12	1.06 ± 0.09
Claudin-12 tv2	1.73 ± 0.21*	1.72 ± 0.19*	1.96 ± 0.08*	1.17 ± 0.13	1.00 ± 0.00	1.07 ± 0.13	1.27 ± 0.09*^§^	1.87 ± 0.19*	1.98 ± 0.28*
Claudin-12 tv3	1.17 ± 0.16	1.26 ± 0.13	1.31 ± 0.04*	1.14 ± 0.12	1.00 ± 0.00	0.95 ± 0.10	0.99 ± 0.08^§^	1.63 ± 0.14*	1.49 ± 0.24
Claudin-14	1.22 ± 0.14	0.77 ± 0.14	0.84 ± 0.13	1.01 ± 0.07	1.00 ± 0.00	1.16 ± 0.29	0.75 ± 0.08*	0.99 ± 0.16	0.52 ± 0.07*^§^
Claudin-15	1.01 ± 0.13	0.90 ± 0.12	1.77 ± 0.47	1.17 ± 0.07*	1.00 ± 0.00	1.41 ± 0.34	0.81 ± 0.12^§^	1.22 ± 0.14	1.88 ± 0.22*^§^
Claudin-16	1.81 ± 0.28*	1.62 ± 0.32	1.25 ± 0.20	0.99 ± 0.11	1.00 ± 0.00	1.07 ± 0.21	0.70 ± 0.05*^§^	1.08 ± 0.11	0.66 ± 0.12*^§^
Claudin-17	12.78 ± 8.63	11.44 ± 8.08	35.16 ± 24.62	4.33 ± 3.99	1.00 ± 0.00	5.63 ± 3.59	20.47 ± 18.10	2.14 ± 0.57	119.56 ± 83.89
Claudin-18 tva	1.16 ± 0.26	1.07 ± 0.23	2.97 ± 1.20	1.09 ± 0.13	1.00 ± 0.00	1.97 ± 0.70	0.23 ± 0.07*	0.31 ± 0.08*	0.88 ± 0.39
Claudin-18 tvb	1.53 ± 0.57	3.03 ± 1.25	4.07 ± 2.79	0.61 ± 0.27	1.00 ± 0.00	0.77 ± 0.25	1.89 ± 1.37	1.07 ± 0.33	8.96 ± 5.38
Claudin-20	1.24 ± 0.17	1.19 ± 0.27	2.04 ± 0.59	0.66 ± 0.13*	1.00 ± 0.00	1.77 ± 0.49	0.25 ± 0.10*	0.38 ± 0.12*	0.63 ± 0.22
Claudin-22	0.81 ± 0.11	0.85 ± 0.08	1.18 ± 0.16	0.99 ± 0.10	1.00 ± 0.00	1.14 ± 0.17	0.62 ± 0.12*	0.93 ± 0.14	1.57 ± 0.45
Claudin-23	0.82 ± 0.08*	0.99 ± 0.07	0.92 ± 0.21	0.80 ± 0.15	1.00 ± 0.00	0.70 ± 0.17	0.64 ± 0.15*	0.95 ± 0.16	1.72 ± 0.76
Claudin-24	0.87 ± 0.15	0.98 ± 0.28	1.51 ± 0.31	1.05 ± 0.13	1.00 ± 0.00	1.62 ± 0.36	0.57 ± 0.17*	0.81 ± 0.13	2.34 ± 0.98
Claudin-25	3.47 ± 1.94	8.17 ± 4.46	8.08 ± 5.39	1.04 ± 0.65	1.00 ± 0.00	1.77 ± 0,76	4.02 ± 2.93	3.39 ± 0.14	22.29 ± 14.18
ZO-1	0.80 ± 0.15	0.65 ± 0.06*	1.16 ± 0.42	1.20 ± 0.20	1.00 ± 0.00	1.78 ± 0.56	0.86 ± 0.11	1.53 ± 0.33	1.62 ± 0.33
ZO-2	0.81 ± 0.08*	0.93 ± 0.08	1.26 ± 0.17	0.99 ± 0.09	1.00 ± 0.00	1.16 ± 0,19	0.86 ± 0.08^§^	1.39 ± 0.14*	1.61 ± 0.21*
ZO-3	1.21 ± 0.24	2.12 ± 0.70	3.81 ± 0.96*	1.37 ± 0.39	1.00 ± 0.00	3.39 ± 0.72*	0.79 ± 0.21^§^	1.71 ± 0.30*	2.80 ± 0.42*
JAM-1	0.90 ± 0.07^#^	1.27 ± 0.11*	1.42 ± 0.08*	0.94 ± 0.12	1.00 ± 0.00	1.14 ± 0.18	0.56 ± 0.05*^§^	0.80 ± 0.07*	0.87 ± 0.12
JAM-2	0.69 ± 0.31	1.23 ± 0.24	1.92 ± 0.42*	1.45 ± 0.70	1.00 ± 0.00	0.84 ± 0.23	0.79 ± 0.13	1.30 ± 0.21	0.54 ± 0.13*^§^
JAM-3	0.91 ± 0.12	1.43 ± 0.39	1.13 ± 0.11	1.05 ± 0.13	1.00 ± 0.00	0.94 ± 0.07	0.84 ± 0.08	1.13 ± 0.11	1.42 ± 0.22
Occludin	0.89 ± 0.10	0.78 ± 0.06*	1.23 ± 0.29	1.00 ± 0.12	1.00 ± 0.00	1.67 ± 0.54	0.85 ± 0.10^§^	1.28 ± 0.15	1.23 ± 0.15
Tricellulin	1.00 ± 0.07	1.21 ± 0.11	2.56 ± 0.98	0.84 ± 0.09	1.00 ± 0.00	1.56 ± 0.40	0.74 ± 0.05*^§^	1.05 ± 0.07	1.27 ± 0.14
CDH5	0.81 ± 0.09	0.83 ± 0.03*	1.04 ± 0.26	0.94 ± 0.09	1.00 ± 0.00	1.11 ± 0.27	0.80 ± 0.05*^§^	1.23 ± 0.09*	1.05 ± 0.14
ABCB1 (PgP)	0.82 ± 0.11	0.79 ± 0.06*	1.81 ± 0.53	1.30 ± 0.16	1.00 ± 0.00	1.61 ± 0.50	1.01 ± 0.09	1.07 ± 0.13	1.41 ± 0.19
ABCC1 (MRP1)	1.28 ± 0.16	1.00 ± 0,10	2.31 ± 0.96	1.20 ± 0.10	1.00 ± 0.00	1.88 ± 0.71	1.13 ± 0.19	1.27 ± 0.09*	1.48 ± 0.16*
ABCC2 (MRP2)	1.22 ± 0.12^#^	0.94 ± 0.03*	1.45 ± 0.37	1.25 ± 0.14	1.00 ± 0.00	1.45 ± 0.60	0.99 ± 0.07	1.13 ± 0.12	1.04 ± 0.16
ABCC3 (MRP3)	0.91 ± 0.10	0.91 ± 0.06	2.19 ± 0.16^#^*	0.85 ± 0.09	1.00 ± 0.00	1.94 ± 0.41*	0.66 ± 0.07*	0.72 ± 0.04*	1.65 ± 0.28*^§^
ABCC4 (MRP4)	0.65 ± 0.06*	0.68 ± 0.02*	1.33 ± 0.30	1.01 ± 0.09	1.00 ± 0.00	1.32 ± 0.37	0.55 ± 0.05*	0.59 ± 0.06*	0.74 ± 0.12
ABCC5 (MRP5)	1.27 ± 0.21	1.14 ± 0.07	2.54 ± 0.90	1.22 ± 0.13	1.00 ± 0.00	1.52 ± 0.50	1.11 ± 0.11	1.45 ± 0.15*	1.88 ± 0.24*
ABCG2 (BCRP)	1.04 ± 0.10	1.14 ± 0.08	1.48 ± 0.16*	1.01 ± 0.10	1.00 ± 0.00	1.20 ± 0.26	1.04 ± 0.07	1.28 ± 0.15	1.49 ± 0.25
SLC2A1 (Glut1)	1.41 ± 0.16^#^*	0.85 ± 0.07*	0.85 ± 0.15	1.00 ± 0.10	1.00 ± 0.00	1.19 ± 0.25	2.62 ± 0.36*	3.63 ± 0.42*	4.09 ± 0.51*
VEGFa	2.93 ± 0.50*	1.26 ± 0.14	1.25 ± 0.36	1.06 ± 0.11	1.00 ± 0.00	1.29 ± 0.40	4.46 ± 0.62*	5.63 ± 0.49*	5.83 ± 0.57*

Data were normalized on β-actin (n = 8 from four independent experiments). Claudin-2 und—19 were not detected, claudin-13 and -21 are not present in human, tv = transcript variant. Data are presented as mean ± SEM of the x-fold regulation related to hCMEC/D3 cells, which were cultivated from day 5 to 6 in EBM-2 with 0.25% FCS, their medium was changed on day 6 in DMEM plus glucose and treated for 5 h under normoxic conditions. Statistically significant regulations were marked with: *p < 0.05 versus 0.25% EBM-2 (day 5 to 6) and DMEM + Glu (5 h treatment) and N (normoxia); ^#^p < 0.05 versus 0.25% EBM-2 (day 5 to 6) and 0.25% EBM-2 (5 h treatment) and N (normoxia); ^§^p < 0.05 versus 0.25% EBM-2 (day 5 to 6) and DMEM—Glu (5 h treatment) and OGD (oxygen/glucose deprivation

In order to elucidate whether specific treatments and targets were regulated in clusters together, a hierarchical cluster analysis of the data from the nine treatments was performed (Fig. 3). With regard to the treatments it was found that samples in the same cultivation media under normoxic conditions (EBM-2 media normoxia, 5 and 0.25% FCS = Norm_Ctrl 5% 6d, Norm_Ctrl 0.25% 6d; DMEM (+ glucose) normoxia precultured in EBM-2 = Norm_Gluc_5% 6d, Norm_Gluc_0.25% 6d) clustered together. Also the samples treated with hydrocortisone clustered, suggesting that the serum reduction per se was a weaker regulator than the medium exchange. With regard to the targets several clusters were identified proposing a similar regulation behavior for claudin-20 with claudin-18 tv 1b, JAM-1 with JAM-2 and ABCC4, ABCC2 with claudin-7, claudin-12 tv1 and claudin-16, ABCC1 with ABCC5 and tricellulin, ABCB1 with claudin-15, occludin and ZO-1, Jam-3 with claudin-12 tv3 and ABCG2, ZO-2 with CDH5 and claudin-22, ABCC3 with claudin-3, claudin-4 and claudin-24, claudin-1 with claudin-11 and claudin-14, claudin-5 with claudin-6, claudin-12 tv2, claudin-18 tv2a and ZO-3, VEGFa with SLC2A1 and claudin-8 with claudin-9, claudin-10 tv a, claudin-10 tv b, claudin-17 and claudin-25.

### Influence of co-cultivation with glioma C6 cells on functional barrier breakdown and target expressions

To investigate the influence of glioma C6 cells co-treated with hCMEC/D3 in the Transwell model, it was decided to cultivate the hCMEC/D3 cells for 5 days and reduce the serum content from 5 to 0.25% from day 5 to day 6 in order to induce the paracellular barrier. Hydrocortisone was not added due to its anti-inflammatory properties [14] and dominant effects shown above, which might bias the elucidation of the sole effects of C6 cells. Differences of raw electrical resistance values between cell and blank inserts before starting OGD experiments were for hCMEC/D3 in monoculture subjected to DMEM—glucose 14.0 ± 4.6 Ω, for hCMEC/D3 in monoculture subjected to DMEM + glucose 15.5 ± 3.9 Ω for hCMEC/D3 in co-culture with rat glioma C6 cells subjected to DMEM + glucose 14.2 ± 1.5 Ω and for hCMEC/D3 in co-culture with rat glioma C6 cell subjected to DMEM—glucose 13.1 ± 6.4 Ω. Five hours of OGD treatment at 1% O_2_ resulted in no effect on TEER in the hCMEC/D3 mono-culture, whereas the co-culture with C6 cells led to a significant decrease to 36 ± 6% (Fig. 4). Corresponding to this, the permeability of the paracellular marker FITC-dextran 4000 (FD4) was increased to 978 ± 161% by the co-culture with C6 cells. Interestingly, the permeability data also revealed an influence of the co-culture with C6 cells under normoxic conditions (235 ± 26%) and sole OGD (493 ± 115%).

HCMEC/D3 cells were lysed immediately after the experiment and were analysed by qPCR. No statistically significant effect was found comparing hCMEC/D3 mono- and co-culture with C6 cells under normoxic conditions, whereas OGD increased significantly the expression of claudin-1 (3.06 ± 0.21), claudin-5 (1.70 ± 0.10), claudin-12 tv2 (1.77 ± 0.12), claudin-12 tv3 (1.66 ± 0.15), claudin-15 (3.27 ± 0.42), claudin-16 (1.45 ± 0.11), claudin-24 (1.60 ± 0.35), ZO-1 (1.74 ± 0.16), ZO-2 (2.29 ± 0.18), occludin (2.15 ± 0.22), CDH5 (1.43 ± 0.13) in comparison to mono-culture normoxic samples (1.00 ± 0.00), but decreased the expression of claudin-3 (0.29 ± 0.09) and claudin-7 (0.78 ± 0.06) (Additional file 2). With regard to the other targets ABCB1 (1.82 ± 0.31), ABCC1 (1.45 ± 0.11), ABCC5 (1.79 ± 0.04) and VEGFa (2.38 ± 0.49) were significantly upregulated after OGD in comparison to the mono-culture normoxic samples (1.00 ± 0.00), whereas ABCC4 (0.67 ± 0.03) was significantly downregulated. Similar effects were found when comparing co-culture OGD with co-culture normoxia samples, except that in this case also claudin-11 (0.58 ± 0.07) and JAM-1 (0.83 ± 0.05) were downregulated after OGD. Interestingly, the co-cultivation with C6 cells led to a less pronounced upregulation of hypoxia marker VEGFa in comparison to the mono-culture OGD. Further detailed data and the statistical analysis between the OGD treatments in mono- versus co-culture could be found in Additional file 2. Figure 5 depicted the hierarchical cluster analysis of the according qPCR results. With regard to the treatment, a clear distinction was found between normoxia and OGD samples. Cluster analysis revealed following target clusters: Jam-2 with claudin-16, ABCC1 with claudin-22 and claudin-24, CDH5 with claudin-6 and Jam-3, ABCG2 with claudin-12 tv1 and tricellulin, ABCC5 with claudin-14, ABCB1 and ZO-1, claudin-12 tv2 with claudin-12 tv3 and occludin, claudin-7 with claudin-11, claudin-20, Jam-1, ABCC3 and ABCC4, claudin-4 with claudin-18 tv 1b and claudin-23, claudin-5 with claudin-9, claudin-1 with claudin-15 and ZO-2, VEGFa with SLC2A1, claudin-8 with claudin-10 tv a, claudin-10 tv b, claudin-17, claudin-18 tv 2a and claudin-25.

### Influence of co-cultivation with primary human astrocytes and pericytes on functional barrier breakdown and target expressions

Preliminary experiments with co-culture of hA and hP revealed that reduction to 1% O_2_ was not sufficient to achieve the aimed barrier damage between 35 and 60%. Therefore, following experiments were carried out at 0.1% O_2_. Differences of raw electrical resistance values between cell and blank inserts before starting OGD experiments were for hCMEC/D3 in monoculture subjected to DMEM—glucose 19.4 ± 2.9 Ω, for hCMEC/D3 in monoculture subjected to DMEM + glucose 12.4 ± 2.5 Ω, for hCMEC/D3 in co-culture with hA/hP subjected to DMEM + glucose 11.5 ± 2.5 Ω and for hCMEC/D3 in co-culture with hA/hP subjected to DMEM—glucose 18.1 ± 2.3 Ω. Data showed that OGD alone already decreased TEER to 56 ± 3% and the co-cultivation even further reduced TEER to 39 ± 2%. Corresponding to the TEER data, FITC-dextran 4000 permeability increased mostly to 205 ± 35% after the co-culture with astrocytes and pericytes, but OGD had no significant effect (124 ± 15%) in comparison to the normoxic mono-culture control (Fig. 6). qPCR analysis of the collected hCMEC/D3 samples after the experiments showed that the presence of astrocytes/pericytes decreased the expression of claudin-22 (0.74 ± 0.11) and claudin-24 (0.69 ± 0.18) under normoxic conditions (Additional file 3). OGD treatment led to significantly increased claudin-12 tv 1 (1.59 ± 0.25) and occludin (1.71 ± 0.12), but to a decrease of mRNA levels of claudin 18 tv1b (0.50 ± 0.18), claudin-22 (0.79 ± 0.08) and JAM-1 (0.76 ± 0.02) compared to normoxia samples. Co-cultivation with astrocytes/pericytes revealed an upregulation of claudin-15 (1.37 ± 0.26) after OGD in comparison to co-cultured hCMEC/D3 cells under normoxic conditions (0.76 ± 0.05). In case of other targets OGD treatment of mono-cultured hCMEC/D3 significantly decreased ABCC3 (0.64 ± 0.13) and ABCC4 (0.63 ± 0.02) mRNA expression and increased SLC2A1 (3.40 ± 1.10) when compared to mono-culture normoxia samples (1.00 ± 0.00). Further detailed data analysis is listed in Additional file 3. With regard to the hierarchical cluster analysis again the normoxia samples as well as the OGD samples grouped together. With regard to the targets following clusters were identified (Fig. 7): ABCC3 with claudin-4, JAM-1, claudin-3, JAM-3 with ZO-1, claudin-7, claudin-14 with ABCC5, tricellulin with ABCG2, JAM-2 with claudin-20 and claudin-16, claudin-15 with claudin-1, ZO-2 with claudin-12 tv1 and occludin, CDH5 with claudin-6, claudin-12 tv2 with claudin-12 tv3 and ABCB1, ABCC2 with claudin-18 tv2 and claudin-5, claudin-25 with claudin-17, ABCC4 with claudin-18 tv 1b, claudin-24 with claudin-11 and claudin-22, VEGFa with SLC2A1 and claudin-10 tva with claudin-8.

## Discussion

The study has three main aspects. First, a human BBB cerebral ischemia in vitro model should be developed in which the desired paracellular damage to 35–60% can be achieved in at least 6 h. This was considered to be very essential in order to be able to realistically simulate the course of functional BBB damage observed in in vivo models and in the clinic [41]. Secondly, by using the standard growth medium for the hCMEC/D3 cells, a medium change to another basal medium for OGD treatment is necessary (from EBM-2 to DMEM), since no glucose-free EBM-2 medium is available by default. In order to better interpret the data from the OGD experiments, it is necessary to check to what extent the change to a distinctly different basal medium has a relevant influence on the final results. In addition, the influence of common cultivation variants for hCMEC/D3 prior to the OGD experiment was tested on the expression of barrier relevant targets. Thirdly, there has been a discussion for a long time which tight junction proteins, especially which claudins, occur at the BBB in general, but also species-specifically. The investigation on the protein level is very difficult because there is a lack of specific antibodies for all claudins due to their high sequence homologies. For this reason, we have decided to conduct a comprehensive study on the transcript level in which all human claudins 1–25 were analyzed together with other TJ and BBB relevant ABC transporters in the most different conditions. Since first publications showed that the compensation of claudins can play an important role in the post stroke regeneration process [42] and that data on jointly regulated clusters of these targets are hardly known, it was decided to perform hierarchical cluster analysis. The obtained data can then be applied to propose possibly first coherent regulatory clusters. For these analyses we have deliberately opted for qPCR as the analytical method, as this has the widest dynamic range, the lowest quantification limits and the least biased results in comparison to microarrays or RNA-seq analysis [6, 13, 15]. This is especially important for TJ proteins as they are often regulated within a small range of 0.5 to twofold.

In recent years, in vitro BBB models for cerebral ischemia based on mouse brain endothelial cells have shown that co-culture was essential for a significant barrier damage within a few hours. Both rat glioma cell line C6 and primary glial cells were applied in these mouse models using 0–1% O_2_ [29, 31]. The selected oxygen range of 0–1% is physiologically relevant considering the fact that almost no oxygen is left in the ischemic core (less than 5 mm Hg pO_2_) after 1 h cerebral ischemia in vivo [25]. Preliminary experiments with hCMEC/D3 showed that incubation with glioma C6 cells for 5 h could be sufficient for the desired barrier collapse. Since the paracellular barrier of hCMEC/D3 cells is generally rather moderate, it is important to note that methodically the use of Corning Costar inserts is advantageous for this type of studies due to the slits that define spatially the measuring point for the chopstick electrodes, and the higher porosity and thus lower inherent electrical resistance of the porous membranes. For the Transwell studies hCMEC/D3 were incubated as mono- and co-cultures for 5 h under normoxia and OGD conditions. Cell viability of hCMEC/D3 was not detrimentally reduced under these conditions (Additional file 4) which was also confirmed by others [36, 53]. In this context, it was previously shown that it was necessary to apply 16–24 h OGD at 1% O_2_ to reduce cell viability and TEER of mono-cultures of hCMEC/D3 to 5–20% or of hiPSC-BCECs (hiPSC = derived from human induced pluripotent stem cells) to 20–50% of the respective normoxia controls [21, 36, 37, 53]. Moreover, hCMEC/D3 mono-cultures treated with OGD at 1% O_2_ for 6 h resulted in slight, but not significant changes of TEER and fluorescein permeability compared to the 6 h incubated normoxia control [37]. These data together with ours confirmed that no reasonable BBB breakdown was achievable without co-cultures in the aimed time window of maximum 6 h. Co-cultivation of hCMEC/D3 with rat glioma C6 cells resulted in the aimed barrier breakdown after 5 h of OGD treatment at 1% O_2_. In the case of co-cultivation with the 1:1 mixture of primary, human astrocytes/pericytes, in contrast to 1% O_2_, clear barrier damage was achieved at 0.1% O_2_ after 5 h (effects at 1% O_2_, see Additional file 5). Noteworthy, the integrity of the mono-cultures were also significantly disrupted under these conditions. The difference between the effects of the co-cultures with C6 and the primary human cells could be explained by the different amounts of secreted growth factors, since the C6 cells secrete e.g. significant more barrier damaging VEGF than primary astrocytes [4, 52].

As first step for the comprehensive barrier target analysis the relative expression of these targets was assessed in hCMEC/D3 under standard growth conditions. In the BBB research field the claudins-1, -3, -5, -11 and -12 are mostly investigated as surrogate markers for TJs, whereas recent studies with knock-out mice raised doubts about the presence and role of claudin-3 and -12 at the BBB [7, 8]. In the last 2 to 3 years some reports with expression data of primary human BCECs and hiPSC-BCECs have been published. These confirmed the expression of almost all claudins in human BBB in vitro models [11, 24, 47]. However, these data were mostly obtained from RNA-seq analysis and were not validated by a second method, so there is still a need to validate them with e.g. qPCR. For example, an open-accessible, but yet not peer reviewed report claims that the hiPSC-BCECs do not express claudin-5, although this has already been shown by many other groups [26]. This may be due to the general problem of lab-to-lab reproducibility of experiments and in particular by the lab-to-lab reproducibility of hiPSC differentiation protocols as well as the lack of validation of these expression data. Nevertheless, these data indicate that the human BBB may contain significantly more claudins than have been researched in detail so far. In order to classify the expression data of the hCMEC/D3 cells obtained in this work, Table 2 shows a comparison of these data with data from human isolated brain capillaries [3] and mouse brain endothelial cells from two RNAseq databases [46, 54]. The most strongly expressed claudins in the mouse brain endothelium found in both databases were claudin-5, claudin-12 and claudin-6. In addition, claudin-2, claudin-3, claudin-16, claudin-18, claudin-20 and claudin-22 were also listed in both databases. In case of the human capillary samples the most prominent claudins were claudin-5, claudin-11, claudin-12 and claudin-1. In comparison to these data, hCMEC/D3 cells expressed most claudin-11, claudin-1, claudin-12 and claudin-10. Interestingly, claudin-11 was not found in the mouse databases, whereas it was very strong in the human capillaries as well as in hCMEC/D3 cells. In this regard, Berndt et al. [3] proved the expression of claudin-11 also in mouse brain capillaries by qPCR indicating how important it is to validate RNAseq data by a second method. The most striking differences to the other samples was that hCMEC/D3 showed a high expression of claudin-10 and relatively low expression of claudin-5. In this context, the role of claudin-10 in hCMEC/D3 would still need to be investigated. According to Berndt et al. [3] claudin-10 is a paracellular pore forming claudin. It could be speculated, whether the knock-down of claudin-10 in hCMEC/D3 might increase paracellular tightness of hCMEC/D3 layers. The low amount of claudin-5 in hCMEC/D3 cells and the probably associated moderate paracellular barrier function were already shown by several groups [12] and confirmed by low TEER values in the present study. Recently, it was shown that the overexpression of claudin-5 in hCMEC/D3 cells led to a reduction in the permeation of A549 cancer cells by approximately two-thirds, but did not lead to a noticeable improvement in electrical impedance. This suggested that additional mechanisms were missing to functionally incorporate claudin-5 at the tight junctions in the cell membrane [27]. Single cell RNA-seq data of mouse brain derived cells from the Betsholtz group [46] showed that fibroblast-like cells and not brain capillary endothelial cells expressed claudin-1. This led to the question whether claudin-1 is present at the BBB and how relevant it is. In contrast to these data, claudin-1 was found in RNA-seq data of mouse brain endothelial cells by Zhang et al. [54]. Moreover, human brain capillaries and hCMEC/D3 cells showed significant expression of claudin-1 (Table 2). In this regard, a recent study showed that claudin-1 replaces claudin-5 at the TJ of brain capillary endothelial cells during the regeneration phase after stroke. This replacement was associated with a weaker barrier [42]. Considering these data it could be speculated whether the strong expression of claudin-1 in the hCMEC/D3 may also be partly responsible for the weaker barrier of hCMEC/D3 cell layers. Finally, it must be mentioned that Berndt et al. [3] reported that the gene Cldnd1 was expressed in human brain capillaries as the strongest claudin, even significantly more than claudin-5. Cldnd1 was also strongly expressed in mouse brain endothelial cells and seems to have sealing properties [3, 54]. It has to be mentioned that the cell line hCMEC/D3 was originally immortalized from brain endothelial cells isolated from resected brain tissue from an epileptic patient [50]. Thus, it cannot be totally excluded that still disease specific properties are present in this cell line. This could be e.g. relevant for ABC transporter profiles, since it is known that the transporter ABCB1 can be upregulated during epilepsy [22]. The comparison of the expression of ABC transporters in hCMEC/D3 under standard conditions with mRNA and proteomics data from isolated capillaries showed that only ABCB1, ABCG2 and ABCC4 were detected at the protein level in human brain capillaries, whereas on the mRNA level ABCB1, ABCC5, ABCG2, ABCC1 and ABCC4 could be found in this expression order (ABCC2 and ABCC3 were not measured on the mRNA level in the brain capillaries [23, 40, 45]). Analysis of hCMEC/D3 samples showed that all tested ABC transporters of this work were also found in previous publications, but partly in different order (mRNA: ABCB1, ABCC3, ABCC1, ABCC4, ABCC5 and very little ABCC2, [48]; protein: ABCB1, ABCG2, ABCC1, ABCC4 (ABCC2, 3 and 5 under detection limit); [35]. This may also have been due to the fact that a different basal growth medium was used in the compared publication or barrier regulating supplements such as hydrocortisone were already present in the medium [48]. For this reason, the effects of switching the medium from EBM-2 to DMEM for the OGD experiment were also investigated as a parameter of transcript analysis. In this regard, it is known that EBM-2 basal medium contains significantly less glucose than the used DMEM (1.2 versus 4.5 g glucose/L) and that ABC transporters can be regulated by different glucose levels [39]. Moreover, it was shown recently that sole medium exchange in the controls of OGD experiments led to a significant decrease of paracellular tightness of a BBB in vitro model based on primary endothelial cells [44]. In addition, the influence of serum reduction and the addition of hydrocortisone was included in the study as both treatments were used to increase the low claudin-5 content and thus the paracellular barrier of hCMEC/D3 layers [14]. The mRNA expression data were then not only used to learn about the relative regulations dependent on the different conditions, they were also subjected to hierarchical cluster analysis (Figs. 3, 5 and 7) to identify more general regulation patterns. In this case, the differences in experimental designs should be not neglected such as the type of growth surface (well plates—Fig. 3; Transwell inserts—Fig. 5 and 7) or the amount of data points (Fig. 3 > than Figs. 5 or 7). However, some general observations could be made. The hypoxia markers VEGFa and SLC2A1 clustered together in every of the three experimental series as expected and confirmed the reliability of the applied method. Interestingly, several target pairs clustered together only in both Transwell experimental set-ups, for example, claudin-1 with claudin-15, claudin-17 with claudin-25, ABCC3 with JAM-1, ABCG2 with tricellulin and CDH5 with claudin-6. On the contrary, ABCC3 clustered with claudin-3 and claudin-4 in the co-culture Transwell experiment with human primary astrocytes/pericytes (Fig. 7) as well as in the well-plate experiment (Fig. 2) just like claudin-5 with claudin-18 tv2a, whereas ABCB1 clustered together with ZO-1 in the co-culture set-up with glioma C6 cells (Fig. 5) and the well-plate experiment (Fig. 2). Interestingly, the clustering of claudin-3 with claudin-4, claudin-5 with claudin-18 and CDH5 with claudin-6 is concerning paracellular sealing proteins, whereas the clustering of claudin-17 with claudin-25 represented a claudin pair which is known for paracellular pore-formation [3]. In case of the cluster pair ABCG2 and tricellulin it was shown for both targets that they were regulated under hypoxic or OGD conditions [9, 44].

**Table 2:** Comparison of claudin expression data in brain endothelial cells or capillaries derived from the present work (hCMEC/D3), from Berndt et al. [3] (laser-microdissected human brain capillaries), the Barres^1^ (isolated brain endothelial cells) and the Betsholtz data base^2^ (single cell sequences brain capillary endothelial cells—capEC)

Species	Current work qPCR^3^	Rank	Berndt et al. [3] qPCR^4^	Rank	Barres data base (FPKM)	Rank	Betsholtz data base (counts)	Rank
	Human		Human		Mouse		Mouse	
Cldn1	1000	2	30	4	0.2	7	nd	–
Cldn2	nd	–	1.26	15	0.1	11	3	4
Cldn3	5.2	20	6.08	9	0.3	5	0.05	9
Cldn4	18	16	4.43	11	0.1	11	nd	–
Cldn5	39	9	205	1	< 3500	1	1500	1
Cldn6	18	15	7.51	7	2	3	0.1	7
Cldn7	219	5	nd	–	0.12	9	nd	–
Cldn8	18	14	nd	–	0.1	11	nd	–
Cldn9	25	12	13.4	5	0.15	8	nd	–
Cldn10	142, 116	6^a^	nd	–	nd	–	nd	–
Cldn11	3189	1	162	2	nd	–	nd	–
Cldn12	281, 18, 291	3, 4, 13^b^	114	3	5	2	20	3
Cldn13	#	–	#	–	0.1	11	nd	–
Cldn14	69	7	nd	–	nd	–	nd	–
Cldn15	17	17	6.13	8	0.4	4	nd	–
Cldn16	8	19	nd	–	0.1	11	0.02	10
Cldn17	30	10	5.85	10	0.1	11	nd	–
Cldn18	27, 3.71	11, 22^c^	nd	–	0.1	11	0.1	7
Cldn19	nd	–	nd	–	0.1	11	< 0.1	11
Cldn20	52	8	3.35	13	0.1	11	0.4	5
Cldn21	nd	–	nd	–	##	–	##	–
Cldn22	9.97	18	3.95	12	0.3	5	0.2	6
Cldn23	0.25	24	1.59	14	0.1	11	nd	–
Cldn24	3.65	23	nd	–	0.12	9	na	–
Cldn25	4.77	21	nd	–	na	–	30	2

nd = not detected; CLDN21 = CLDN22 at human; na = not available; #: not encoded in human; ##: not encoded in mouse; ^a^data listed are for Cldn10 tva and tvb; ^b^data listed are for Cldn12 tv1, tv2, tv3; ^c^data listed are for Cldn18 tv1b and tv2a.; ^1^Zhang et al. [54], accessed on 21st of January 2020: https://web.stanford.edu/group/barres_lab/brain_rnaseq.html; ^2^Vanlandewijck et al. [46], He et al. [16], accessed on 21st of January 2020: http://betsholtzlab.org/VascularSingleCells/database.html; ^3^mean values related to Cldn1, which was set to 1000; ^4^mean values * 10^−4^ from the supplementary file of Berndt et al. [3]

Although claudin-1 and claudin-5 were not found in the same cluster of the first level, these two major sealing proteins were located in quite near clusters (Figs. 3 and 5) indicating possible connected regulations. In this regard, Sladojevic et al. (2018) reported that claudin-1 was embedded in the TJ complex after stroke and counteracted regeneration processes by inhibiting the renewal of claudin-5 within the TJ structure. In summary, it was shown that hierarchical cluster analysis could be used to suggest novel regulatory relationships of barrier targets, but also highlighted that single experimental manipulations can have a significant impact on the outcome. Further in-depth studies have to be conducted to investigate and validate the found relationships on a molecular level.

## Conclusion

The first conclusion of this study is that micro-environmental cells significantly strengthen the functional breakdown in a human BBB in vitro model of cerebral ischemia and shift the necessary duration of OGD treatments into a more in vivo-relevant time window. Based on the data, it could be recommended to use the co-culture model with the C6 cells for therapeutic screening applications, since the barrier breakdown is stronger in this model. For purely biological basic questions the model with the primary astrocytes/pericytes should be preferred. If studies on the influence of glioblastoma cells on the BBB during cerebral ischemia were the goal, one should consider using human glioblastoma rather than rat glioma C6 cells for the co-culture model. Future studies could deal with the secretome in the two co-culture models to better understand the different effects of C6 cells and the astrocyte/pericyte mixture on the barrier. The second conclusion is that different cultivation methods (serum reduction, hydrocortisone addition) prior to OGD treatment have a significant effect on the results and regulation of barrier targets. Therefore, it is essential to test the influence of these parameters and consider them in the experimental design. The third conclusion is that hierarchical cluster analysis can be a very helpful tool to elucidate regulated gene clusters, to find counter-regulations and to understand relevant parameters for the experimental design.

## Supplementary information

**Additional file 1.** Agarose gel of PCR products for several claudins expressed in hCMEC/D3 cells.

**Additional file 2.** Regulation of mRNA expression of barrier markers in hCMEC/D3 cells in mono- or co-culture with rat glioma C6 cells after OGD treatment

**Additional file 3.** Regulation of mRNA expression of barrier markers in hCMEC/D3 cells in mono- or co-culture with astrocytes and pericytes after OGD treatment

**Additional file 4.** Influence of OGD treatment (0.1% O_2_, 5 h) on cell viability of mono-cultured hCMED/D3 cells on Transwell inserts

**Additional file 5.** Influence of five hours OGD (1% O_2_) on barrier functionality of hCMEC/D3 in mono-culture and in co-culture with astrocytes and pericytes

## Data Availability

The datasets used and/or analysed during the current study are available from the corresponding author on reasonable request.

## Abbreviations

ABC: ATP binding cassette
BBB: Blood–brain barrier
BCEC: Brain capillary endothelial cell
Claudin: CLDN
FCS: Fetal calf serum
FD4: FITC dextran 4000
hA: Human astrocyte
hbFGF: Human basic fibroblast growth factor
hiPSC: Human induced pluripotent stem cell
hP: Human pericyte
JAM: Junctional adhesion molecule
OGD: Oxygen/glucose deprivation
PBS: Phosphate buffered saline
P-l-L: poly-l-lysine
qPCR: Quantitative polymerase chain reaction
RNA-seq: RNA-sequencing
TEER: Transendothelial electrical resistance
TJ: Tight junction
tv: Transcript variant
VEGF: Vascular endothelial growth factor
ZO: Zonula occludens
