# Supplementary material for: The pivotal role of micro-environmental cells in a human blood–brain barrier in vitro model of cerebral ischemia: functional and transcriptomic analysis
Source: Fluids Barriers CNS. 2020 Mar 5;17:19. doi: 10.1186/s12987-020-00179-3 (PMC7059670; doi:10.1186/s12987-020-00179-3)
Supplement: Supplementary file 1 — Additional file 1. Agarose gel of PCR products for several claudins expressed in hCMEC/D3 cells. [file 12987_2020_179_MOESM1_ESM.pdf]

Additional file 1

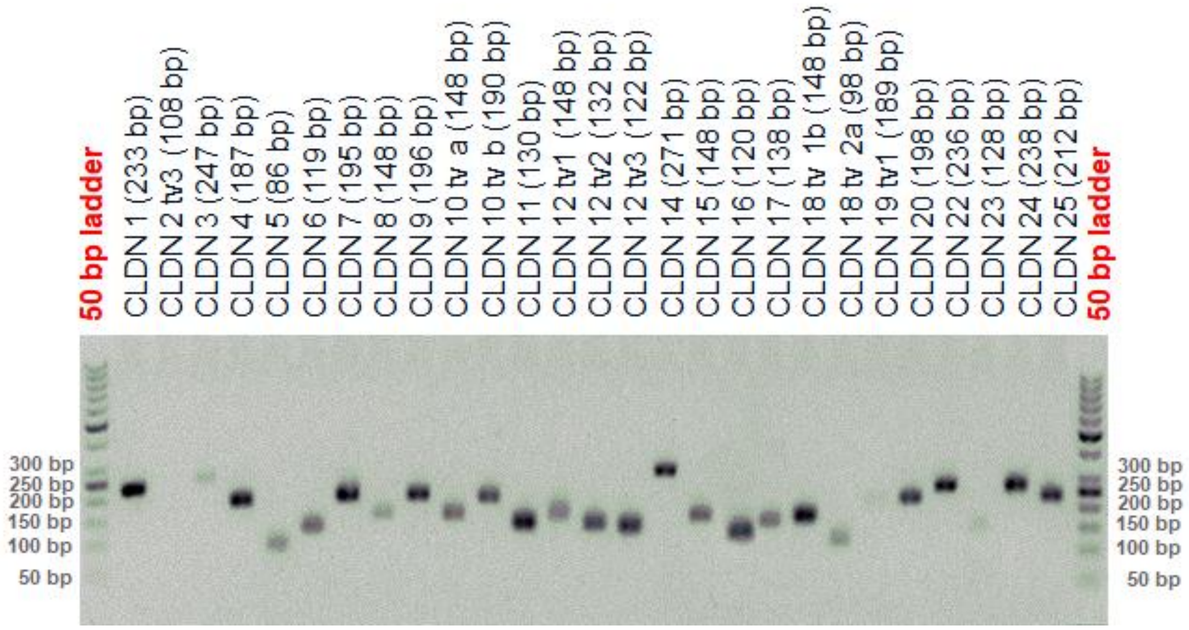

Figure S1: Agarose gel of PCR products for several claudins (CLDN) expressed in hCMEC/D3 cells, claudin-13 and -21 do not exist in human, claudin-2 and claudin-19 showed no significant expression in hCMEC/D3.
