# Supplementary material for: The pivotal role of micro-environmental cells in a human blood–brain barrier in vitro model of cerebral ischemia: functional and transcriptomic analysis
Source: Fluids Barriers CNS. 2020 Mar 5;17:19. doi: 10.1186/s12987-020-00179-3 (PMC7059670; doi:10.1186/s12987-020-00179-3)
Supplement: Supplementary file 2 — Additional file 2. Regulation of mRNA expression of barrier markers in hCMEC/D3 cells in mono- or co-culture with rat glioma C6 cells after OGD treatment [file 12987_2020_179_MOESM2_ESM.pdf]

Additional file 2

Table S1: Regulation of mRNA expression of barrier markers in hCMEC/D3 cells in mono- or co-culture with rat glioma C6 cells after OGD treatment (1% O_2, 5 h) in DMEM - glucose or after subjection to DMEM + glucose in normoxia for 5 hours. Relative x-folds of mRNA expression normalized to mono-cultured hCMEC/D3 in normoxia are illustrated. Data presented as means ± SD, N=3, n=3 (pooling of four replicates in each different experiment was performed). As endogenous control β-actin was used. Calculated x-folds are shown as means ± standard deviation (SD). Statistical significance were indicated with *: $p<0.05$ *versus* hCMEC/D3 + C6 normoxia, #: $p<0.05$ *versus* hCMEC/D3 OGD and §: $p<0.05$ *versus* hCMEC/D3 normoxia, one way-ANOVA and following Tukey post-hoc test, or Kruskal-Wallis one way-ANOVA on ranks if normal distribution of data set was not given. tv = transcript variant.

Day 5	EBM-2 0.25% FCS			
**Day 6 – 5hr**	**hCMEC/D3 DMEM + glucose normoxia**	**hCMEC/D3 + C6 DMEM + glucose normoxia**	**hCMEC/D3 DMEM - glucose OGD**	**hCMEC/D3 + C6 DMEM - glucose OGD**
**CLDN1**	1.00 ± 0.00	1.05 ± 0.11	3.06 ± 0.21§*	1.86 ± 0.28§*#
**CLDN3**	1.00 ± 0.00	0.45 ± 0.24	0.29 ± 0.09§	1.16 ± 0.47*#
**CLDN4**	1.00 ± 0.00	0.95 ± 0.19	0.83 ± 0.07	1.09 ± 0.26
**CLDN5**	1.00 ± 0.00	0.80 ± 0.14	1.70 ± 0.10§*	2.29 ± 0.11§*#
**CLDN6**	1.00 ± 0.00	1.08 ± 0.38	1.37 ± 0.17	1.37 ± 0.18
**CLDN7**	1.00 ± 0.00	0.89 ± 0.06	0.78 ± 0.06§	0.72 ± 0.05§*
**CLDN8**	1.00 ± 0.00	1.52 ± 1.04	3.98 ± 4.04	5.40 ± 3.54
**CLDN9**	1.00 ± 0.00	1.21 ± 0.38	2.08 ± 1.26	3.25 ± 0.90§
**CLDN10 tva**	1.00 ± 0.00	1.22 ± 1.07	3.70 ± 3.36	5.41 ± 3.60
**CLDN10 tvb**	1.00 ± 0.00	2.55 ± 1.31	4.59 ± 5.46	7.06 ± 7.26
**CLDN11**	1.00 ± 0.00	0.97 ± 0.06	0.94 ± 0.17	0.58 ± 0.07§*#
**CLDN12 tv1**	1.00 ± 0.00	0.96 ± 0.17	1.26 ± 0.26	1.06 ± 0.11
**CLDN12 tv2**	1.00 ± 0.00	1.02 ± 0.08	1.77 ± 0.12§*	1.48 ± 0.11§*#
**CLDN12 tv3**	1.00 ± 0.00	1.09 ± 0.22	1.66 ± 0.15§*	1.58 ± 0.10§*
**CLDN14**	1.00 ± 0.00	1.05 ± 0.60	1.85 ± 0.97	1.22 ± 0.75
**CLDN15**	1.00 ± 0.00	0.97 ± 0.14	3.27 ± 0.42§*	1.82 ± 0.62#
**CLDN16**	1.00 ± 0.00	0.79 ± 0.19	1.45 ± 0.11§*	0.70 ± 0.15#
**CLDN17**	1.00 ± 0.00	1.72 ± 1.02	5.47 ± 7.00	6.45 ± 4.94
**CLDN18 tv1b**	1.00 ± 0.00	1.18 ± 0.35	0.93 ± 0.07	1.03 ± 0.46
**CLDN18 tv2a**	1.00 ± 0.00	1.78 ± 1.29	3.77 ± 3.03	4.11 ± 2.32
**CLDN20**	1.00 ± 0.00	0.98 ± 0.18	1.11 ± 0.24	0.75 ± 0.08
**CLDN22**	1.00 ± 0.00	1.11 ± 0.22	1.45 ± 0.24	1.10 ± 0.19
**CLDN23**	1.00 ± 0.00	1.09 ± 0.29	1.11 ± 0.46	1.06 ± 0.42
**CLDN24**	1.00 ± 0.00	0.95 ± 0.17	1.60 ± 0.35§*	1.12 ± 0.23
**CLDN25**	1.00 ± 0.00	2.17 ± 1.28	6.66 ± 8.34	7.25 ± 5.78
**ZO-1**	1.00 ± 0.00	1.21 ± 0.16	1.74 ± 0.16§*	1.34 ± 0.09§#
**ZO-2**	1.00 ± 0.00	1.12 ± 0.16	2.29 ± 0.18§*	1.85 ± 0.13§*#
**Occludin**	1.00 ± 0.00	1.28 ± 0.20	2.15 ± 0.22§*	1.25 ± 0.12#
**CDH5**	1.00 ± 0.00	1.07 ± 0.13	1.43 ± 0.13§*	1.23 ± 0.10
**JAM-1**	1.00 ± 0.00	1.05 ± 0.05	0.85 ± 0.10*	0.83 ± 0.05§*
**JAM-2**	1.00 ± 0.00	1.24 ± 0.65	1.45 ± 0.94	0.80 ± 0.30
**JAM-3**	1.00 ± 0.00	0.98 ± 0.31	1.25 ± 0.28	1.25 ± 0.15
**Tricellulin**	1.00 ± 0.00	0.84 ± 0.08	1.27 ± 0.14*	1.05 ± 0.16
**ABCB1**	1.00 ± 0.00	1.19 ± 0.13	1.82 ± 0.31§*	1.32 ± 0.16#
**ABCC1**	1.00 ± 0.00	1.07 ± 0.07	1.45 ± 0.11§*	1.08 ± 0.13#
**ABCC2**	1.00 ± 0.00	1.04 ± 0.15	1.33 ± 0.06	1.59 ± 0.27§*
**ABCC3**	1.00 ± 0.00	1.03 ± 0.10	0.71 ± 0.15*	0.62 ± 0.13§*
**ABCC4**	1.00 ± 0.00	1.19 ± 0.13	0.67 ± 0.03§*	0.52 ± 0.09§*
**ABCC5**	1.00 ± 0.00	1.09 ± 0.25	1.79 ± 0.04§*	1.31 ± 0.11#
**ABCG2**	1.00 ± 0.00	0.94 ± 0.04	1.37 ± 0.29*	0.97 ± 0.09
**SLC2A1**	1.00 ± 0.00	0.98 ± 0.04	5.10 ± 0.77*	1.79 ± 0.10
**VEGFa**	1.00 ± 0.00	0.90 ± 0.14	4.78 ± 0.96*	2.20 ± 0.26
