# Supplementary material for: The pivotal role of micro-environmental cells in a human blood–brain barrier in vitro model of cerebral ischemia: functional and transcriptomic analysis
Source: Fluids Barriers CNS. 2020 Mar 5;17:19. doi: 10.1186/s12987-020-00179-3 (PMC7059670; doi:10.1186/s12987-020-00179-3)
Supplement: Supplementary file 3 — Additional file 3. Regulation of mRNA expression of barrier markers in hCMEC/D3 cells in mono- or co-culture with astrocytes and pericytes after OGD treatment [file 12987_2020_179_MOESM3_ESM.pdf]

Additional file 3

Table S2: Regulation of mRNA expression of barrier markers in hCMEC/D3 cells in mono- or co-culture with astrocytes and pericytes after OGD treatment (0.1% O_2, 5 h) in DMEM - glucose or after subjection to DMEM + glucose in normoxia for 5 hours. Relative x-folds of mRNA expression normalized to mono-cultured hCMEC/D3 in normoxia are illustrated. Data presented as means ± SD, N=3, n=3 (pooling of four replicates in each different experiment was performed). As endogenous control β-actin was used. Calculated x-folds are shown as means ± standard deviation (SD). Statistical significance were indicated with *: $p<0.05$ *versus* hCMEC/D3 + hA/hP normoxia, #: $p<0.05$ *versus* hCMEC/D3 OGD and §: $p<0.05$ *versus* hCMEC/D3 normoxia, one way-ANOVA and following Tukey post-hoc test, or Kruskal-Wallis one way-ANOVA on ranks if normal distribution of data set was not given. tv = transcript variant.

Day 5			EBM-2 0.25% FCS	
**Day 6 – 5hr**	**hCMEC/D3 DMEM + glucose normoxia**	**hCMEC/D3 + hA/hP DMEM + glucose normoxia**	**hCMEC/D3 DMEM - glucose OGD**	**hCMEC/D3 + hA/hP DMEM - glucose OGD**
**CLDN1**	1.00 ± 0.00	0.64 ± 0.10	1.31 ± 0.59	1.14 ± 0.26
**CLDN3**	1.00 ± 0.00	1.13 ± 0.46	0.55 ± 0.14*	0.90 ± 0.61
**CLDN4**	1.00 ± 0.00	0.94 ± 0.13	0.63 ± 0.04*	0.87 ± 0.19
**CLDN5**	1.00 ± 0.00	0.66 ± 0.13	0.99 ± 0.26	1.60 ± 0.65
**CLDN6**	1.00 ± 0.00	0.76 ± 0.13	1.08± 0.09	1.12 ± 0.26
**CLDN7**	1.00 ± 0.00	0.92 ± 0.12	0.89 ± 0.04	0.87 ± 0.09
**CLDN8**	1.00 ± 0.00	1.33 ± 1.07	2.17 ± 2.79	8.15 ± 13.14
**CLDN9**	1.00 ± 0.00	0.62 ± 0.06	0.67 ± 0.30	0.87 ± 0.52
**CLDN10 tva**	1.00 ± 0.00	1.03 ± 0.66	1.07 ± 1.07	4.99 ± 7.45
**CLDN10 tvb**	1.00 ± 0.00	0.45 ± 0.18	0.48 ± 0.24	1.25 ± 1.78
**CLDN11**	1.00 ± 0.00	0.77 ± 0.18	0.67 ± 0.18	0.52 ± 0.10§
**CLDN12 tv1**	1.00 ± 0.00	0.86 ± 0.17	1.59 ± 0.25§*	1.30 ± 0.21
**CLDN12 tv2**	1.00 ± 0.00	0.92 ± 0.18	1.26 ± 0.07	1.16 ± 0.19
**CLDN12 tv3**	1.00 ± 0.00	0.88 ± 0.09	1.16 ± 0.27	1.15 ± 0.09
**CLDN14**	1.00 ± 0.00	0.80 ± 0.37	0.83 ± 0.28	0.87 ± 0.14
**CLDN15**	1.00 ± 0.00	0.68 ± 0.14	1.53 ± 0.44*	1.39 ± 0.29
**CLDN16**	1.00 ± 0.00	0.61 ± 0.09	1.20 ± 0.51	0.77 ± 0.19
**CLDN17**	1.00 ± 0.00	0.86 ± 0.57	0.96 ± 0.31	2.66 ± 3.65
**CLDN18 tv1b**	1.00 ± 0.00	0.83 ± 0.22	0.50 ± 0.18§	0.54 ± 0.25
**CLDN18 tv2a**	1.00 ± 0.00	0.75 ± 0.34	0.75 ± 0.31	1.72 ± 2.23
**CLDN20**	1.00 ± 0.00	0.78 ± 0.31	1.04 ± 0.10	0.70 ± 0.21
**CLDN22**	1.00 ± 0.00	0.74 ± 0.11§	0.79 ± 0.08§	0.60 ± 0.05§#
**CLDN23**	1.00 ± 0.00	0.95 ± 0.32	0.79 ± 0.46	1.03 ± 0.59
**CLDN24**	1.00 ± 0.00	0.69 ± 0.18§	0.77 ± 0.14	0.47 ± 0.07§
**CLDN25**	1.00 ± 0.00	0.90 ± 0.63	0.82 ± 0.40	2.06 ± 2.11
**ZO-1**	1.00 ± 0.00	0.88 ± 0.02	0.96 ± 0.09	0.86 ± 0.09
**ZO-2**	1.00 ± 0.00	0.90 ± 0.11	1.55 ± 0.43*	1.25 ± 0.15
**Occludin**	1.00 ± 0.00	0.88 ± 0.17	1.71 ± 0.12§*	1.13 ± 0.03#
**CDH5**	1.00 ± 0.00	0.82 ± 0.19	1.13 ± 0.25	1.26 ± 0.17
**JAM-1**	1.00 ± 0.00	1.05 ± 0.10	0.76 ± 0.02§*	0.85 ± 0.14
**JAM-2**	1.00 ± 0.00	0.72 ± 0.22	0.95 ± 0.53	0.78 ± 0.22
**JAM-3**	1.00 ± 0.00	0.83 ± 0.09	0.95 ± 0.10	0.90 ± 0.13
**Tricellulin**	1.00 ± 0.00	0.87 ± 0.10	1.06 ± 0.14	0.96 ± 0.13
**ABCB1**	1.00 ± 0.00	1.00 ± 0.00	1.18 ± 0.50	1.19 ± 0.35
**ABCC1**	1.00 ± 0.00	0.99 ± 0.10	0.97 ± 0.18	0.88 ± 0.03
**ABCC2**	1.00 ± 0.00	0.68 ± 0.12	0.76 ± 0.25	1.36 ± 0.52
**ABCC3**	1.00 ± 0.00	0.98 ± 0.18	0.64 ± 0.13§	0.79 ± 0.13
**ABCC4**	1.00 ± 0.00	0.98 ± 0.12	0.63 ± 0.02§*	0.57 ± 0.05*§
**ABCC5**	1.00 ± 0.00	0.85 ± 0.20	0.87 ± 0.05	0.80 ± 0.14
**ABCG2**	1.00 ± 0.00	1.01 ± 0.07	1.08 ± 0.20	1.00 ± 0.13
**SLC2A1**	1.00 ± 0.00	0.82 ± 0.16	3.40 ± 1.10§*	1.81 ± 0.53
**VEGFa**	1.00 ± 0.00	0.74 ± 0.32	2.64 ± 1.38	1.87 ± 0.83
