# Supplementary material for: The pivotal role of micro-environmental cells in a human blood–brain barrier in vitro model of cerebral ischemia: functional and transcriptomic analysis
Source: Fluids Barriers CNS. 2020 Mar 5;17:19. doi: 10.1186/s12987-020-00179-3 (PMC7059670; doi:10.1186/s12987-020-00179-3)
Supplement: Supplementary file 4 — Additional file 4. Influence of OGD treatment (0.1% O2, 5 h) on cell viability of mono-cultured hCMED/D3 cells on Transwell inserts [file 12987_2020_179_MOESM4_ESM.pdf]

Additional file 4

Cell viability test

hCMEC/D3 cells were seeded at a density of 80,000 cells/cm^2 onto collagen IV/fibronectin coated Transwell inserts. On day 6 after cell seeding, hypoxia and OGD experiments (0.1% O_2, 5% CO_2, 37 °C) were performed in a hypoxic chamber (Biospherix, USA) for 5 hours. The control plate was subjected to normoxic conditions (air atmosphere, 5% CO_2, 37 °C). After TEER measurement medium was changed in a part of the inserts with DMEM with glucose. According to the manufacturer's protocol 20 µL/insert of the dissolved EZ4U reagent (1:10 of the stock solution) were added and cells were incubated for two hours in the incubator (air atmosphere, 5% CO_2, 37 °C, 95% humidity). Cell viability was measured in technical duplicates by using a microplate reader (EnSpire® Multimode Plate Reader, Waltham, USA). Absorption was measured at wavelengths 450 nm with a background measurement at 620 nm, cell viability was calculated in [%] after substraction of blank media related to the normoxia control.

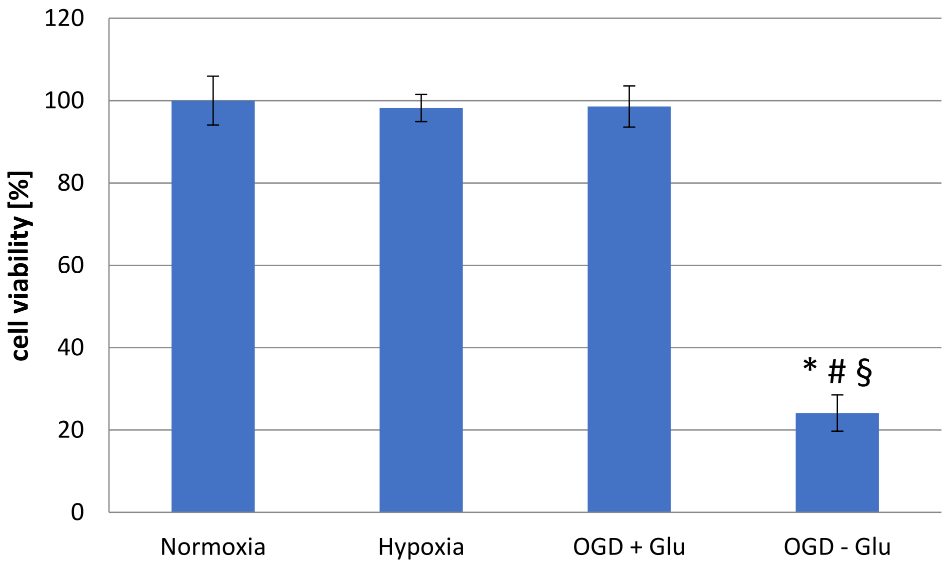

Figure S2: Influence of OGD treatment (0.1% O_2, 5 hours) on cell viability of mono-cultured hCMED/D3 cells on Transwell inserts. Normoxia: hCMEC/D3 cells in DMEM + glucose under normoxic conditions. Hypoxia: hCMEC/D3 cells in DMEM + glucose were subjected to hypoxic conditions. OGD + glu: For cell viability studies, medium was changed after OGD treatment from DMEM – glucose to DMEM + glucose. OGD - glu: hCMEC/D3 cells were subjected to OGD treatment and for cell viability studies, medium remained DMEM – glucose which resulted in low conversion of the substrate since glucose was missing (=control for the EZ4U). Data presented as means ± SD, N=3, n=12. Statistical significance ($p < 0.05$, t-test with equal variances) was labeled with * vs. normoxia, # vs. hypoxia and § vs. OGD+Glu.
