# Supplementary material for: The pivotal role of micro-environmental cells in a human blood–brain barrier in vitro model of cerebral ischemia: functional and transcriptomic analysis
Source: Fluids Barriers CNS. 2020 Mar 5;17:19. doi: 10.1186/s12987-020-00179-3 (PMC7059670; doi:10.1186/s12987-020-00179-3)
Supplement: Supplementary file 5 — Additional file 5. Influence of five hours OGD (1% O2) on barrier functionality of hCMEC/D3 in mono-culture and in co-culture with astrocytes and pericytes [file 12987_2020_179_MOESM5_ESM.pdf]

Additional file 5

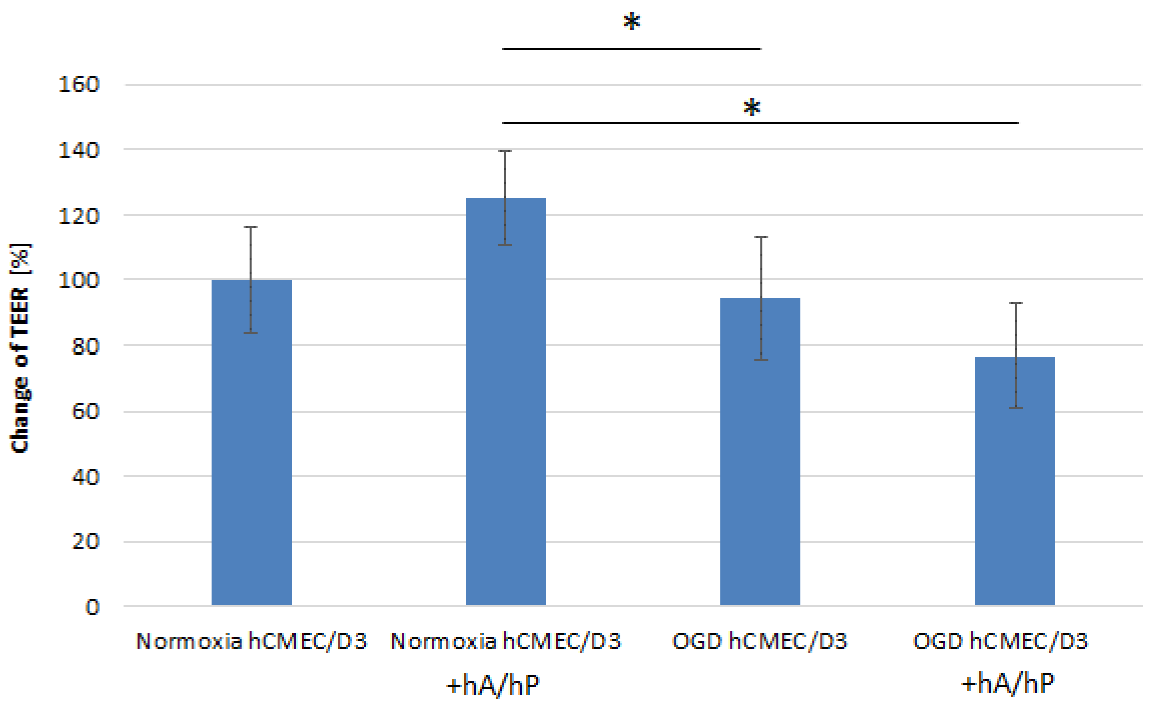

Figure S3: Influence of five hours OGD (1% O_2) on barrier functionality of hCMEC/D3 in mono-culture and in co-culture with astrocytes and pericytes (hA/hP) measured by TEER (n=4 inserts). Data presented as means ± SD. Statistical significance ($p < 0.05$, t-test when equal variances were given, Mann-Whitney rank sum test when no normal distribution of data was given) was labelled with an asterisk (*).
